# Changes in the Composition of Oral and Intestinal Microbiota After Sleeve Gastrectomy and Roux-En-Y Gastric Bypass and Their Impact on Outcomes of Bariatric Surgery

**DOI:** 10.1007/s11695-022-05954-9

**Published:** 2022-02-21

**Authors:** Tomasz Stefura, Barbara Zapała, Tomasz Gosiewski, Oksana Skomarovska, Michał Pędziwiatr, Piotr Major

**Affiliations:** 1 2nd Department of General Surgery, Faculty of Medicine, Jagiellonian University Medical College, Jakubowskiego 2 st, 30-688 Cracow, Poland; 2 Department of Clinical Biochemistry, Faculty of Medicine, Jagiellonian University Medical College, 31-501 Cracow, Poland; 3 Department of Microbiology, Division of Molecular Medical Microbiology, Faculty of Medicine, Jagiellonian University Medical College, 31-121 Cracow, Poland; 4 Centre for Research, Training and Innovation in Surgery (CERTAIN Surgery), 30-688 Cracow, Poland

**Keywords:** Obesity, Bariatric surgery, Microbiota

## Abstract

**Background:**

We aimed to assess the changes in composition of bacterial microbiota at two levels of the digestive tract: oral cavity and large intestine in patients 6 months after bariatric surgery.

**Methods:**

This was a prospective cohort study including patients undergoing bariatric surgery. Before surgery and 6 months after the procedure, oral swabs were obtained and stool samples were provided. Our endpoint was the analysis of the differences in compositions of oral and fecal microbiota prior and after the surgical treatment of obesity.

**Results:**

Bacteria from phylum Bacteroidetes seemed to increase in abundance in both the oral cavity and the large intestine 6 months after surgery among patients undergoing bariatric surgery. The subgroup analysis we conducted based on the volume of weight-loss revealed that patients achieving at least 50% of excess weight loss present similar results to the entire study group. Patients with less favorable outcomes presented an increase in the population of bacteria from phylum Fusobacteria and a decrease of phylum Firmicutes in oral cavity.

**Conclusion:**

Intestinal microbiota among these patients underwent similar changes in composition to the rest of the study group. Bariatric surgery introduces a significant change in composition of oral and intestinal microbiota.

**Graphical abstract:**

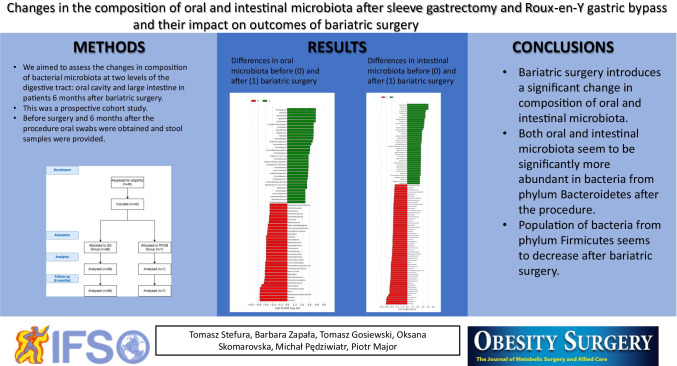

**Supplementary Information:**

The online version contains supplementary material available at 10.1007/s11695-022-05954-9.

## Introduction

The composition of the gut microbiota among people with obesity increases intestinal permeability, which activates the immune system, leading to chronic inflammation thus further increasing the risk of obesity-related comorbidities [1]. It seems that modifying the composition of the intestinal microbiota may be a new strategy in the treatment of obesity.

Currently, bariatric surgery is the most effective method of treating morbid obesity [2–4]. The most frequently performed bariatric surgeries are the laparoscopic sleeve gastrectomy (SG) and the laparoscopic Roux-en-Y gastric bypass (RYGB) [5]. Multiple mechanisms seem to be responsible for the long-term reduction of excess body weight and the improvement of obesity-related diseases [6]. Studies carried out on rats have revealed that bariatric procedures significantly affect the composition of the gastrointestinal microbiota [7].

Most articles published so far describe changes in the microbiota in the biological material collected from the large intestine and concerns studies carried out on animal models [8]. Interference with the anatomy of the gastrointestinal tract during bariatric procedures is mainly related to the upper gastrointestinal tract and varies greatly between SG and RYGB [9]. There are very few studies investigating changes in the microbiota of the gastrointestinal tract among patients treated by SG or studies comparing the results obtained in patients after SG and RYGB. Therefore, further research concerning changes in the microbiota of the oral cavity and large intestine after SG and RYGB surgeries is recommended.

We aimed to assess the changes in composition of bacterial microbiota at two levels of the digestive tract: oral cavity and large intestine in patients 6 months after SG and RYGB — two most performed bariatric surgeries. The secondary aim was to identify potential relationships between those changes and weight-loss outcomes of SG and RYGB.

## Methods

### Study Design

This prospective cohort study was conducted in a teaching hospital, between November 2018 and November 2019. Patients were qualified for bariatric surgery using the following criteria: BMI ≥35 kg/m^2^ with obesity-related comorbidities or BMI ≥40 kg/m^2^ [10]. Inclusion criteria were age between 18 and 65 years old, informed consent to participate in the study, and meeting the eligibility criteria for bariatric treatment, either for SG or RYGB. The choice of SG or RYGB was a consensus reached by a patient and a surgeon. Currently, we lack a precise criterion for qualifying patients for SG or RYGB. Exclusion criteria were treatment with antibiotics within 30 days prior to gathering biological material, treatment with probiotics within 30 days prior to gathering biological material, tooth decay, gastrointestinal infections, inflammatory bowel disease, thyroid diseases, history of cancer (especially in the digestive tract), and immunodeficiency. The study was designed and described regarding all STROBE checklist points for observational studies [11].

The authors created a database concerning patients during bariatric treatment and the follow-up 6 months after the initial bariatric surgery. The database included anthropometric and clinical data: age, sex, preoperative body weight and BMI, maximal BMI, American Society of Anesthesiologists (ASA) class, comorbidities such as type 2 diabetes, hyperlipidemia, steatohepatitis, hypertension, cardiovascular disorders, respiratory disorders, varicose veins and smoking, and factors related to surgical procedures, i.e., type of procedure, operative time, and bariatric treatment parameters 6 months after bariatric surgery: percentage of total body weight loss (%TBWL), percentage of excess weight loss (%EWL), and percentage of excess BMI loss (%EBMIL). Patients were divided into two groups based on weight-loss outcomes achieved 6 months after bariatric surgery (successful (≥50%EWL) and unsuccessful (<50%EWL)). The cut-off point of 50% EWL at 6 months after surgery for successful weight loss was based on previous reports concerning outcomes after bariatric procedures in the short term [12, 13]. We also divided patients based on the type of bariatric surgery into SG and RYGB.

### Analysis of Endpoints

The first endpoint was to analyze the changes in composition of oral and intestinal microbiota 6 months after the SG or RYGB. Secondary endpoint was to identify potential relationship between changes in the composition of oral and intestinal microbiota, and weight-loss outcomes of bariatric surgery.

### Collection and Storage of Swab and Fecal Samples

Patients were advised to fast for at least 12 h prior to gathering the biological material. Swab samples were collected by medical doctors wearing protective clothing and sterile gloves. If patients were wearing dental prosthesis, they were removed prior to taking the oral swab, and oral cavity was rinsed with water. Fecal samples were collected by patients who were informed previously on how to collect samples to minimize the risk of contamination. The swab samples were stored in the original swab collection container without liquid medium. Stool samples were frozen immediately after collection in a −20°C until transport. The samples were transported in a styrofoam container to not increase the temperature. Both stool (after transportation) and oral swab (immediately) samples were stored without DNA stabilizing medium in sterile Eppendorf ® tubes and frozen at −80°C until further processing. According to our previously published protocols of the proceedings, storage at −80°C does not affect the results. Bacterial DNA in this environment remains to be stable and allows for further analysis [14, 15]. All procedures were performed using sterile instruments, ensuring the integrity of the biological material and without undue delay in freezing samples after their collection. The protocol for this study was tested in previous research [16].

### Surgical Technique and Treatment Protocol

An accurate description of the surgical technique used for SG and RYGB in our center is included in our previous publications [17, 18]. The Enhanced Recovery After Surgery (ERAS) pathway was used as a treatment protocol for every patient, including preoperative, intraoperative, and postoperative interventions. It is described in detail in our previous articles [18].

### DNA Isolation, Library Preparation, and Sequencing

Surfaces and equipment were decontaminated with 70% alcohol, and UV radiation was used to minimize environmental contaminants. All consumables used during sample preparation and library preparation were decontaminated by UV treatment. During the bacterial DNA extraction, blank controls were used. Library preparation was performed in a separate room from DNA extraction. During library preparation no-template amplification, controls were included. Filter tips and low aerosol pipettes were used. Additionally, non-redundant dual indexing was performed to prevent index swapping during sequencing. During all sample processing stages, researchers were wearing appropriate clothing including clean laboratory suits, sterile gloves, and face masks. DNA from fecal samples was isolated using QIAamp PowerFecal DNA Kit (QIAGEN) and from swab samples — QIAamp BiOstic Bacteremia DNA Ki (QIAGEN). The quality and quantity of the DNA was assessed using three endpoints. We used the NanoDrop spectrophotometer (Thermofischer) to evaluate DNA purity (A260/280, A260/230), the Qubit fluorometer (Thermofischer) with the 1 X dsDNA HS (high sensitivity) Assay Kit Invitro-gen Q32854) to evaluate DNA yield (ng), and finally Bioanalyzer (Agilent) (DNA 1000). To increase the accuracy and decrease the risk of bias, three negative controls and two positive controls and ATCC standards for oral microbiome (ATCC® MSA-1004™) and gut microbiome (ATCC® MSA-1006™) were included. The V3 and V4 regions (using forward and reverse region-specified primers, selected from Klindworth A. et al. publication) of the 16S rRNA gene were amplified [19]. The PCR was conducted in a 25-ul reaction volume with the following composition: 12.5ul of 2x KAPA HiFi HotStart ReadyMix (ROCHE), 5ul forward and 5ul reverse primer, and 2.5ul (5ng) template. We used 25 cycles of denaturation (95°C for 30 s), annealing (55°C for 30 s), and elongation (72°C for 30 s). To avoid primer-dimer formation, the PCR products were semi-quantified by using Bioanalyzer DNA 1000 chips (Agilent). The index PCR was performed in a 50-ul reaction volume using Nextera XT index kit (FC-131-1001; Illumina). The libraries were validated using the Qubit fluorometer (Thermofischer) with the 1 X dsDNA HS (high sensitivity) Assay Kit (Invitro-gen Q32854) and Bioanalyzer DNA 1000 chips (Agilent). Purified, quantified, and pooled (4nM) amplicons were mixed with 15% of an equimolar concentration of PhiX (Illumina) and sequenced at 5pM. Sequencing was performed with an Illumina MiSeq platform using paired-end 2 × 301 nucleotide (nt) dual-index sequencing.

### Statistical Analysis

Statistical analysis was performed in the STATISTICA v13 package (Tulsa, OK, USA). The data was presented as mean ± standard deviation (SD) — in relation to normal distributions; or as: median (Me) and first (Q1) and third (Q3) quartile — for non-normal distributions. The distribution of the studied variables was verified using the Shapiro–Wilk test. Quantitative data were analyzed with the *T*-student test, Mann–Whitney *U* test, Kruskal–Wallis, ANOVA, and post hoc testing.

We performed the taxonomic classification of 16S rRNA targeted amplicon reads using a taxonomic database. The classification was performed using the Illumina 16S Metagenomics workflow. This analysis was based on the algorithm which is a high-performance implementation of the Ribosomal Database Project (RDP) Classifier described by Wang et al. [20].

To present alpha diversity, we used the Reny index, which depends on the parameter alpha. Alpha=0 gives the total species number, alpha=1 gives an index proportional to the Shannon index, and alpha=2 gives an index that behaves like the Simpson index. The beta-diversity is presented as the principal coordinates analysis, which shows the distances and similarities within the samples.

Linear discriminant analysis (LDA) effect size (LEfSe) is a computational method supporting multidimensional class comparisons, with particular emphasis on metagenomic analyzes. LEfSe identifies the traits (organisms, clades, operational taxonomic units, etc.) that are most likely to explain observed differences. It is achieved by combining standard statistical significance tests with additional tests encoding biological consistency and effect significance. The effect size provided by LEfSe reveals an estimate of the magnitude of the observed difference between previously specified groups [21].

Here, we used LEfSe to identify statistically significant differences in relative abundances of oral microbiota and intestinal microbiota between samples collected prior to and 6 months after bariatric surgery. We have also conducted similar analysis considering specific bariatric operations separately (SG and RYGB) and considering patients achieving successful and unsuccessful outcomes separately.

A default cut-off value of LDA > 2.0 was used in all tests. The Kruskall–Wallis (with alpha value 0.05) test was used to analyze all features, testing whether the values in different classes are differentially distributed. The pairwise Wilcoxon (with alpha value 0.05) was used to verify whether all pairwise comparisons between subclasses within different classes significantly agree with the class level trend. The resulting subset of vectors was used to build a LDA model from which the relative difference among classes is used to rank the features. The final output thus consisted of a list of features that are discriminative with respect to the classes, consistent with the subclass grouping within classes, and ranked according to the effect size with which they differentiate classes. *P*-values below 0.05 (*p* <0.05) were considered to be statistically significant.

## Results

### Demographic Characteristics

**Fig. 1:**
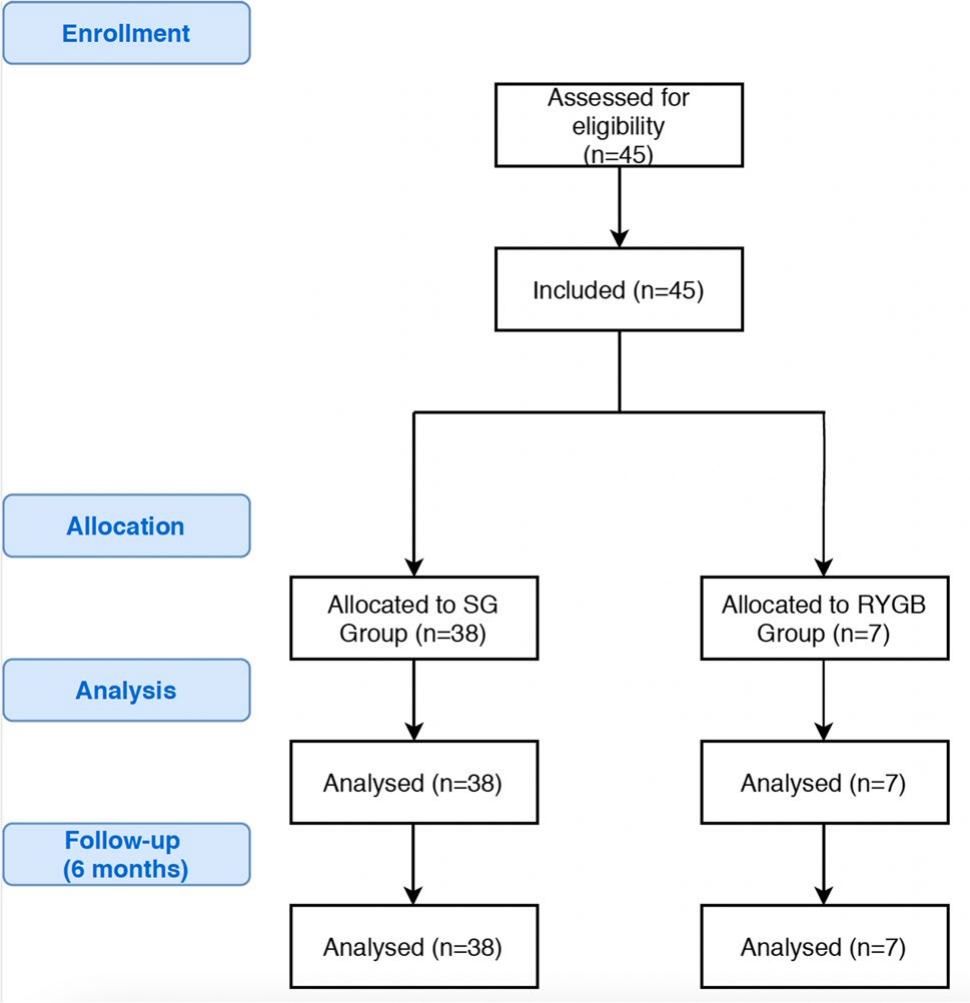
The flow of the study

The study group consisted of 45 patients, 38 (84.4%) underwent SG and 7 (15.6%) underwent RYGB. No participants were lost during the 6-month follow-up period of the study. Oral swabs were collected from all 45 (100%) patients prior to the surgery and during follow-up examinations. We were not able to collect fecal samples from 11 patients during follow-up examination; therefore, analysis of fecal samples was conducted only on 34 patients. A flowchart of the study is presented on Fig. 1. The mean age was 43.5 years ± 11.3 years. Overall, 27 (60%) participants were female. Median maximal weight was 137 kg (126–157 kg), median maximal BMI was 50.3 kg/m^2^ (44.5–54.6 kg/m^2^), and median preoperative BMI was 46.1 kg/m^2^ (41.1–50.2 kg/m^2^). We did not identify significant differences in above mentioned parameters between patients undergoing SG an RYGB. Type 2 diabetes and hyperlipidemia were more frequent in the RYGB group (23.7% vs. 71.4%, *p*=0.012 and 10.5% vs. 57.1%, *p*=0.003). Overall, 37 (82.2%) patients were assigned ASA class II and 8 (17.8%) patients were assigned ASA class III. Median SG operative time was significantly shorter than median RYGB – 95 (75–110) vs. 120 (110–135), *p*=0.009. Additional demographic and perioperative characteristics are presented in Table 1.

**Table 1:** Demographic and perioperative characteristics

Parameter	Total	SG	RYGB	*p*
Total, *n* (%)	45 (100)	38 (84.4)	7 (15.6)	-
Mean age, years ± SD	43.5±11.3	42.5±10.9	48.7±12.5	0.182
Sex (female), *n* (%)	27 (60)	25 (65.8)	2 (28.6)	0.065
Median maximal weight, kg (IQR)	137 (126–157)	136.5 (127–156)	144.5 (124–160)	0.999
Median maximal BMI, kg/m^2^ (IQR)	50.3 (44.5–54.6)	51.5 (44.7–55)	44.8 (43.9–48.4)	0.210
Median preoperative BMI, kg/m^2^ (IQR)	46.1 (41.1–50.2)	46.9 (40.5–50.3)	43.2 (42.3–44.9)	0.253
Type 2 diabetes, *n* (%)	14 (31.1)	9 (23.7)	5 (71.4)	0.012
Hyperlipidemia, *n* (%)	8 (17.8)	4 (10.5)	4 (57.1)	0.003
Steatohepatitis, *n* (%)	8 (17.8)	5 (13.2)	3 (42.9)	0.059
Hypertension, *n* (%)	31 (68.9)	26 (68.4)	5 (71.4)	0.875
Cardiovascular disorders, *n* (%)	8 (17.8)	5 (13.2)	3 (42.9)	0.059
Respiratory disorders, *n* (%)	8 (17.8)	6 (15.8)	2 (28.6)	0.416
Varicose veins, *n* (%)	8 (17.8)	6 (15.8)	2 (28.6)	0.416
Smoking, *n* (%)	5 (11.1)	3 (7.9)	2 (28.6)	0.110
ASA class, *n* (%)				0.416
II	37 (82.2)	32 (84.2)	5 (71.4)	
III	8 (17.8)	6 (15.8)	2 (28.6)	
Median operative time, min. ± SD	100 (80-111.3)	95 (75-110)	120 (110-135)	0.009

*n* Number; *SD* standard deviation; *IQR* interquartile range; *BMI* body mass index; *ASA* American Society of Anesthesiologists

### Outcomes of Bariatric Surgery

After 6 months since surgery, follow-up meetings were carried out with all of the participants. %TBWL, %EWL, and %EBMIL were 29.7±110.1, 53.3±16.6, and 59.5±19.2, respectively. Overall, 26 (57.8%) patients were classified as successful. Additional characteristics concerning the outcomes of bariatric surgery achieved by patients at 6-month follow-up are presented in Table 2.

**Table 2:** Outcomes of bariatric surgery at 6-month follow-up

Parameter	Total	SG	RYGB	*p*
Total, n (%)	45 (100)	38 (84.4)	7 (15.6)	-
%TBWL ± SD	29.7±10.1	30±10.8	28.3±5.1	0.695
%EWL ± SD	53.3±16.6	53.3±17.8	53.2±7.5	0.984
%EBMIL ± SD	59.5±19.2	59.2±20.6	61.3±8.1	0.795
Successful (%EWL>50%)	26 (57.8)	22 (57.9)	4 (57.1)	0.971

*n* Number; *SG* laparoscopic sleeve gastrectomy, *RYGB* Roux-en-Y gastric bypass; *SD* standard deviation; *%TBWL* percentage of total body weight loss; *%EWL* percentage of excess weight loss; *%EBMIL* percentage of excess BMI loss; *BMI* body mass index

### NGS Analysis

We analyzed 158 samples (90 oral swabs and 68 fecal samples) and obtained 18,592,849 reads of the 16S RNA genes. Of these, 16,501,245 (88.7%) passed positive filtering. The mean number of reads per sample was 6659 (range: 4359–39,251). Samples in which the 16S sequence length was <1250 bp, in which there were >50 wobble bases (e.g., M, R, W, S, Y, K, V, H, D, B, or N), or that were only partially classified (no classification for genus or species) were filtered out of the analysis. The cut-off for the number of reads per sample was 2000. There was no need to remove any of the samples from the analysis. The characteristics of the phylogenic analysis of the oral and intestinal samples are presented in Table 3.

**Table 3:** Phylogenetic summary of results obtained

	Taxonomic level	Abundance	Reads PF classified to taxonomic level	% reads PF classified to taxonomic level
Swab samples				
	Kingdom	2	99.429321580645	96.80
	Phylum	57	100.364403225706	98.16
	Class	89	101.081177418322	99.41
	Order	123	101.992838709677	99.33
	Family	336	101.345564516129	98.69
	Genus	1516	102.668664192845	99.31
	Species	3039	81.52847059	79.60
Shanon–Wienner index of diversity	2.87			
Fecal samples				
	Kingdom	2	99.6137736363637	99.66
	Phylum	51	99.4347678982234	99.59
	Class	93	99.1406567689123	99.16
	Order	135	98.0706666456555	99.29
	Family	412	98.3206567676767	98.57
	Genus	1461	93.9656565687654	95.34
	Species	2901	82.5553434345656	82.64
Shanon–Wienner index of diversity	3.53			

*PF* Passed quality filtering

Comparisons of the alpha biodiversity of the oral and intestinal samples are presented in Appendix A. The beta-diversity is presented in Appendix B for the oral (Whittaker index=1.6366) and intestinal (Whittaker index=0.049111) samples.

### Differences in the Microbiota Before and After Bariatric Surgery

**Fig. 2:**
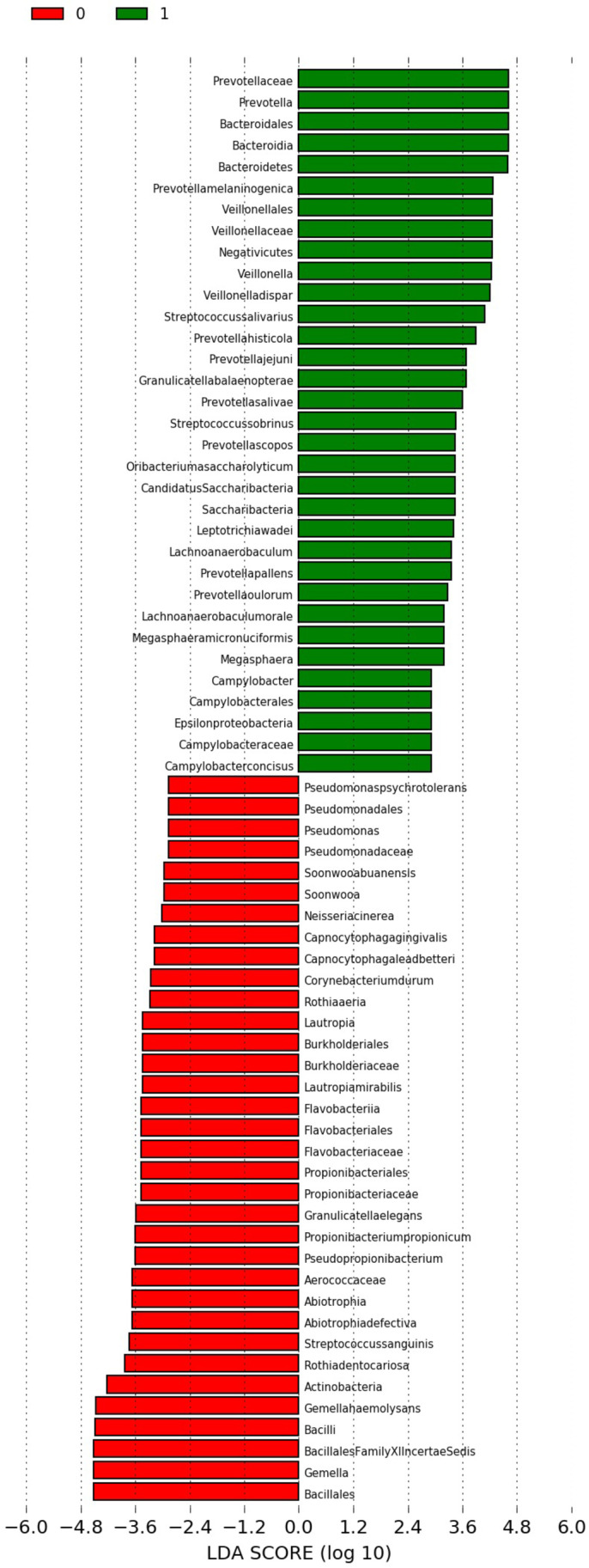
Differences in preoperative and postoperative oral microbiota among patients undergoing bariatric surgery (0 – preoperative microbiota; 1 – postoperative microbiota)

**Fig. 3:**
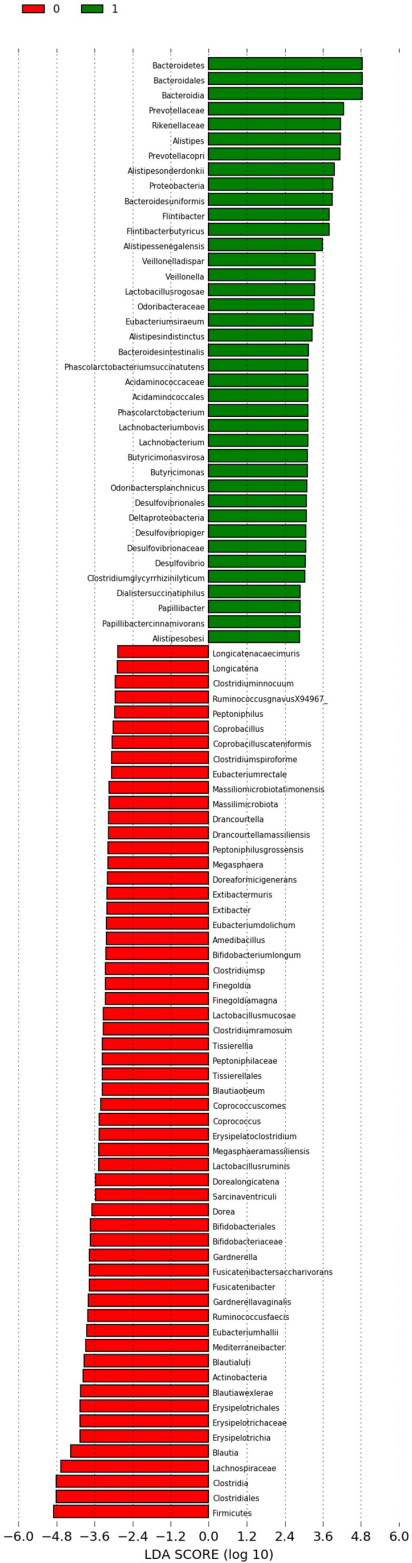
Differences in preoperative and postoperative intestinal microbiota among patients undergoing bariatric surgery (0 – preoperative microbiota; 1 – postoperative microbiota)

According to LEfSe analysis, patients who had undergone bariatric surgery had significantly more bacteria from phylum Bacteroidetes in the oral cavity. Prior to the bariatric procedure, bacteria from phylum Firmicutes were more abundant in the oral cavity (Fig. 2 and Appendix C). Composition of intestinal microbiota after bariatric surgery also included more bacteria from phylum Bacteroidetes. Before bariatric surgery intestinal microbiota were more rich in bacteria, for instance, from phylum Firmicutes (Fig. 3 and Appendix D). The composition (in terms of phyla) of oral and intestinal microbiota in successful and unsuccessful groups before and after the surgery is presented in Appendix E and Appendix F.

### Differences in the Microbiota Before and After Bariatric Surgery in SG Group

When LEfSe analysis was conducted separately on patients undergoing SG, it revealed oral microbiota being most significantly more abundant in phylum Bacteroidetes after surgery and phylum Firmicutes before the bariatric procedure (Appendix G). Intestinal microbiota was observed to be most significantly more abundant in phylum Bacteroidetes after surgery and phylum Firmicutes before the bariatric procedure (Appendix H).

### Differences in the Microbiota Before and After Bariatric Surgery in RYGB Group

Among patients undergoing RYGB oral microbiota was more abundant inter alia in bacteria *Streptococcus salivarius* and bacteria from phylum Bacteroidetes after the operation (Appendix I). Intestinal microbiota among patients undergoing RYGB was more abundant in bacteria from phylum Bacteroidetes after surgery and Clostridiales from phylum Firmicutes before the procedure (Appendix J).

### Differences in the Microbiota Before and After Bariatric Surgery in Successful Group

Patients achieving more than 50% EWL 6 months since bariatric surgery had oral microbiota more abundant in phylum Bacteroidetes after the treatment and *Granulicatella elegans* from phylum Firmicutes before the bariatric surgery (Appendix K). Intestinal microbiota in this group was more plentiful in phylum Bacteroidetes after bariatric treatment and phylum Firmicutes prior to the surgery (Appendix L).

### Differences in the Microbiota Before and After Bariatric Surgery in Unsuccessful Group

Among patients classified as unsuccessful in this study, oral microbiota was significantly more abundant in *Fusobacterium nucleatum* from phylum Fusobacteria after bariatric surgery and *Streptococcus oligofermentans* from phylum Firmicutes before the procedure (Appendix M). Intestinal microbiota after the surgery was more abundant in bacteria from phylum Bacteroidetes in comparison to the composition prior to the surgery, which was more plentiful in bacteria from phylum Firmicutes (Appendix N).

### Changes in the Firmcutes:Bacteroides Ratio After Bariatric Surgery in Successful and Unsuccessful Group

**Fig. 4:**
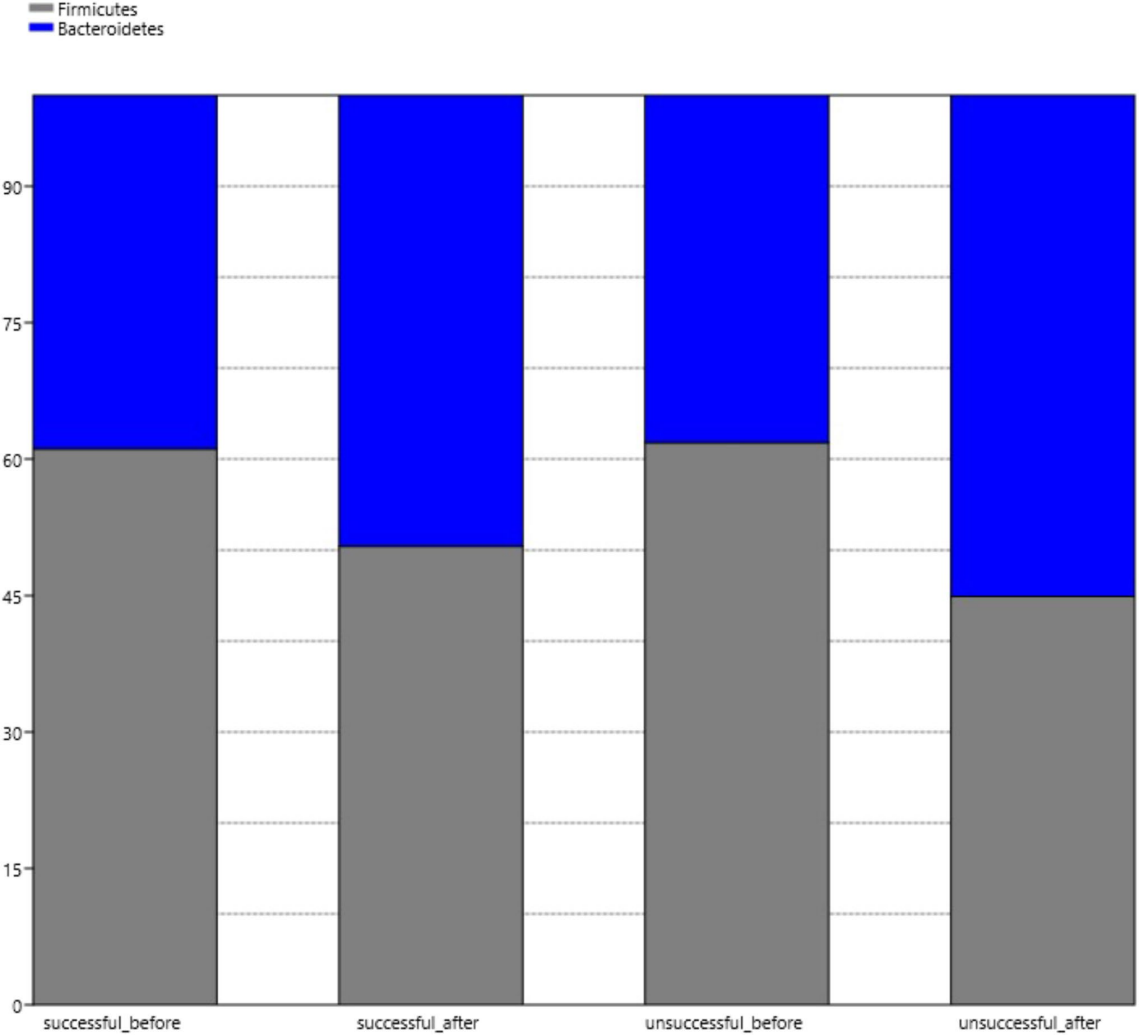
The Firmicutes:Bacteroides ratio before and after bariatric surgery

Among patients achieving favorable outcomes after bariatric surgery the ratio of Firmicutes to Bacteroides before surgery was 61.12 to 38.88%, whereas, after the surgery the ratio was 50.42 to 49.58%. Among patients classified as unsuccessful the ratio of Firmicutes to Bacteroides prior to the operation was 61.79 to 38.21% and after the operation, it shifted to 44.90 to 55.10% (Fig. 4).

## Discussion

This study aimed to identify changes in the composition of oral and intestinal microbiota among patients undergoing SG and RYGB. Size of the study group was relatively abundant when compared with other studies describing microbiota changes occurring after the two, currently most common, bariatric procedures — SG and RYGB [22, 23]. Additionally, we assessed if those changes differ between patients achieving favorable weight loss after bariatric procedure and those with less satisfying outcomes.

To identify the bacterial species, we used a next generation sequencing (NGS) of bacterial 16S RNA. This remains a cutting-edge method for culture-independent analyzing of the quantity of bacterial population in given environment. Currently, NGS is becoming the gold standard in studies investigating human microbiota [24].

*Streptococcus mutans*, *Porphyromonas gingivalis*, *Staphylococcus* sp., and *Lactobacillus* sp. most commonly inhabit the human oral cavity. It is important to remember that the composition of oral microbiota often changes. It is influenced by multiple factors, including lifestyle choices, such as smoking and diet, geography, host genetics, and pregnancy or systemic diseases [25, 26]. Bacterial microbiota in the large intestine also shifts very dynamically. The most common bacteria belong to phyla: Proteobacteria, Firmicutes, Actinobacteria, and Bacteroidetes [27]. Previously identified factors that influence the composition of intestinal microbiota include age, diet, antibiotic use, probiotic use, and systemic diseases [28]. Approximately 3% of patients undergoing bariatric surgery develop symptoms of small intestine bacterial overgrowth (SIBO), which include abdominal pain, bloating, and diarrhea [29]. This highlights the clinical significance of the impact that bariatric surgery has on the composition of bacterial microbiota.

According to our results, bacteria from phylum Bacteroidetes seems to increase in abundance in both the oral cavity and the large intestine 6 months after surgery among patients undergoing bariatric surgery. This increase was observed among patients undergoing SG and RYGB. On the other hand, SG seems to mostly decrease the abundance of bacteria from phylum Firmicutes, whereas RYGB, most significantly reduces the abundance of bacteria from phylum Fusobacteria in the oral cavity and Firmicutes in the large intestine.

A previous study by Furet et al. reported comparable outcomes after RYGB with a significantly increased abundance of phylum Bacteroidetes and reduced abundance of phylum Firmicutes [30]. Also Kong et al., Graessler et al., and Osto et al. reported shifts in bacterial populations after RYGB consistent with our results [31–33]. There are fewer studies reporting microbiota changes after SG. Jahansouz et al. reported an increase of phylum Bacteroidetes and Verrucomicrobia and a decline in phylum Firmicutes following bariatric surgery on mice [34]. A study conducted on rats by Guo et al. reported a higher abundance of Bacteroidetes and a lower of Firmicutes among the SG group compared with the RYGB group [35]. Our results seem to be consistent with majority of previously published research on SG [36]. It is important to notice that majority of available data stems from studies conducted on animal models. We were also unable to find an oral microbiota study with methodology comparable to ours.

The subgroup analysis we conducted, based on the volume of weight loss revealed that patients achieving at least 50% of EWL present similar results to the entire study group. Patients with less favorable outcomes presented an increase in the population of bacteria from phylum Fusobacteria and a decrease of phylum Firmicutes in oral cavity. Intestinal microbiota among these patients underwent similar changes in composition to the rest of the study group.

This study revealed a significant decrease in Firmicutes:Bacteroides ratio in successful as well as in unsuccessful group. Increased Firmicutes:Bacteroides ratio was previously found in patients with obesity and obesity related diseases [37, 38]. This is consistent with previously published studies, which suggest that bariatric surgery may lead to restoration of proper microbial balance in gastrointestinal tract [39]. Unfortunately, the studies presenting the role of Firmicutes:Bacteroides ratio present often conflicting results. Therefore, developing a stratification system for microbiota not only based on taxonomical subgroups might help to conduct more clinically useful studies in the future [40].

Tremaroli et al. reported that transplant of microbiota from patients who successfully underwent bariatric surgery to germ-free mice-induced positive alterations in fat distribution patterns [41]. This suggests that change in the microbiota may constitute an additional mechanism responsible for weight loss occurring after bariatric procedure. Previous studies suggest that intestinal microbiota may influence the weight loss by modulating the bile acids metabolism [42]. Therefore, interventions aiming to influence the gut microbiota may potentially be a useful addition to bariatric surgery, which will aid in achieving improved outcomes.

Unfortunately, the methodology of research on microbiota in bariatric patients differs substantially between the available studies. This often leads to inconsistent results [43]. It is important to include a precise and a comprehensive description of the methodology, including laboratory protocols, when outcomes of microbiota changes are reported [15]. Various factors can influence these outcomes, during different stages of the study [44].

This is one of the first studies that included an analysis of the microbiota in the oral cavity. The reasoning behind conducting this research was that, if microbiota sampling was to enter the clinical environment, it would be more convenient to obtain an oral swab than a fecal sample.

This study has several limitations. The number of patients in the RYGB group is relatively small, although multiple previous studies investigating microbiota changes included a comparable number of patients [22, 23]. Overall, larger study group would yield more precise results. Moreover, difference in size of the SG and RYGB groups introduces potential bias when comparing those groups. Unfortunately, we have reached a limit of the funding granted for this research. Therefore, the results from this group may lack precision and generalizing these results should be done with caution. The article includes subgroup analysis for each procedure, so that results for SG are not skewed. We did not conduct a randomization between SG and RYGB. Therefore, results may be prone to selection bias. Additionally, it is important to consider that observed outcomes may not solely result from the surgery. There are multiple potential factors contributing to changes in the microbiota profile in the six-month course of the study. However, outcomes allow observing a common trend of change in bacterial microbiota composition occurring after the procedure.

Future research should be conducted on a larger sample size, especially studies investigating microbiota changes among patients undergoing RYGB. Preferably studies would be conducted in a multi-center setting and include a double-blind randomization in the study protocol. Including other less popular bariatric procedures such as the single anastomosis gastric bypass, laparoscopic adjustable gastric banding, single anastomosis duodeno-ileal bypass, or biliopancreatic diversion would provide a novel insight into the subject. The authors suggest referencing changes in the microbiota with obesity-related comorbidities, for instance, type 2 diabetes, which was not included in this study protocol due to organizational difficulties with assessing remission or improvement of those ailments. Verifying the stability of the changes of the microbiota over a longer time period requires further research.

## Conclusions

Bariatric surgery introduces a significant change in composition of oral and intestinal microbiota. Both oral and intestinal microbiota seem to be significantly more abundant in bacteria from phylum Bacteroidetes after the procedure, whereas the population of bacteria from phylum Firmicutes seems to decrease after bariatric surgery. Subgroup analysis of microbiota changes among patients achieving satisfying weight loss revealed similar outcomes. Patients classified as unsuccessful in terms of weight loss presented a greater abundance of bacteria from phylum Fusobacteria after surgery. Similar changes with several exceptions are observed independent of the type of surgery (SG vs. RYGB).

## Supplementary Information

Below is the link to the electronic supplementary material.

Supplementary file1 (DOCX 4363 KB)

## Data Availability

The datasets used and/or analyzed during the current study are available from the corresponding author on reasonable request. The funding agencies have no influence on the design of the study and collection, analysis, and interpretation of data and on writing the manuscript.
