# Supplementary material for: Changes in the Composition of Oral and Intestinal Microbiota After Sleeve Gastrectomy and Roux-En-Y Gastric Bypass and Their Impact on Outcomes of Bariatric Surgery
Source: Obes Surg. 2022 Feb 21;32(5):1439–50. doi: 10.1007/s11695-022-05954-9 (PMC8986729; doi:10.1007/s11695-022-05954-9)

Appendix A. Alpha diversity for oral and intestinal microbiota.


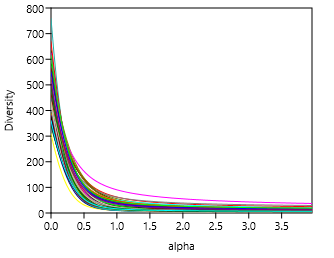


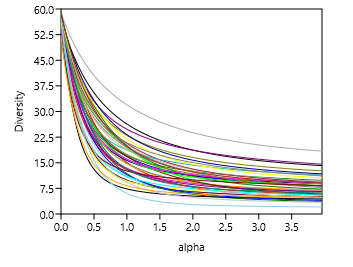


Appendix B. Beta diversity for oral and intestinal microbiota.


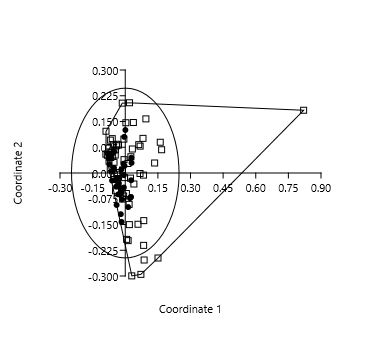


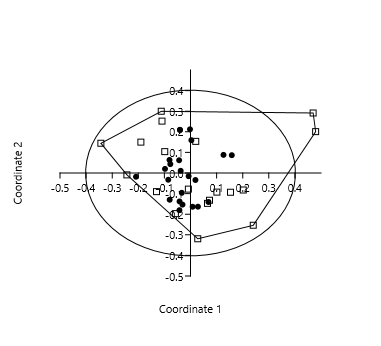


Appendix C. Differences in preoperative and postoperative oral microbiota among patients undergoing bariatric surgery (0 – preoperative microbiota; 1 – postoperative microbiota).


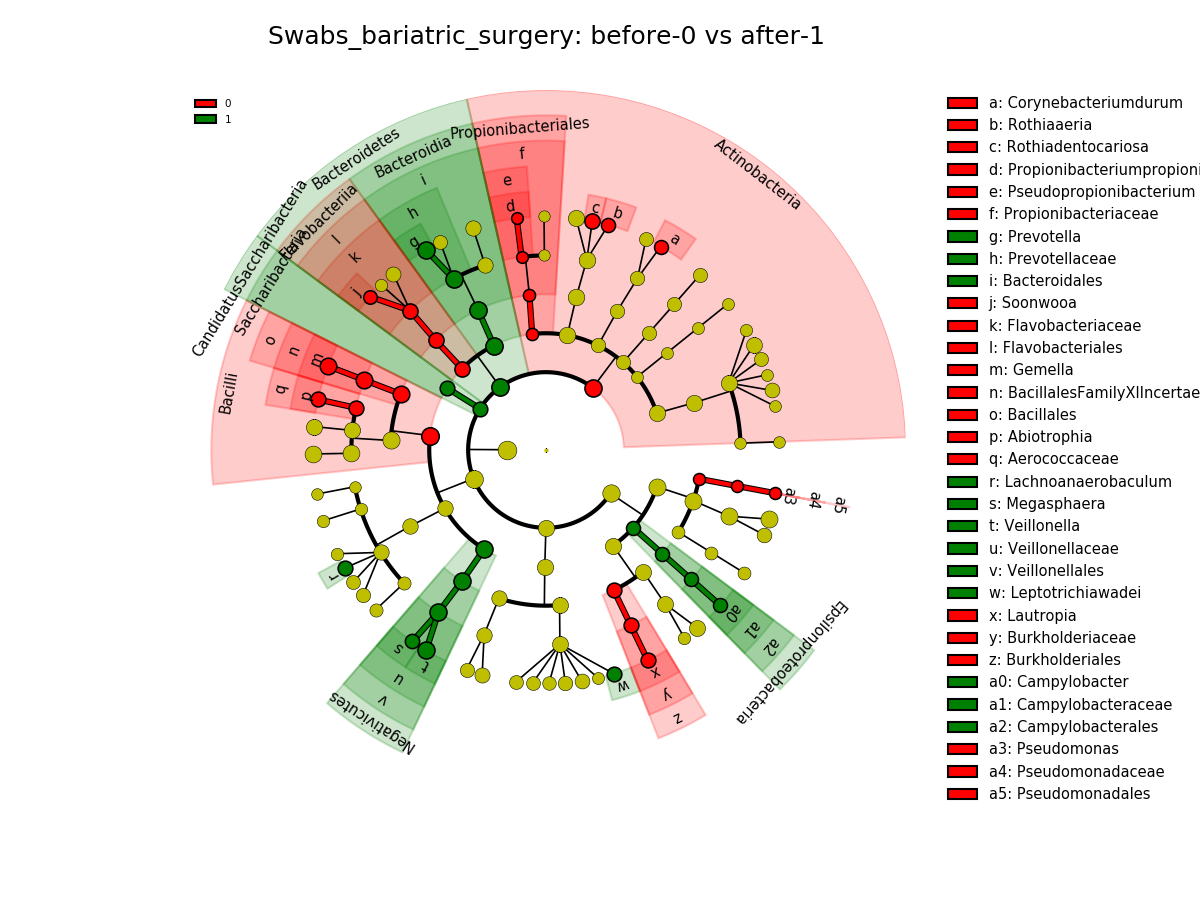


Appendix D. Differences in preoperative and postoperative intestinal microbiota among patients undergoing bariatric surgery (0 – preoperative microbiota; 1 – postoperative microbiota).


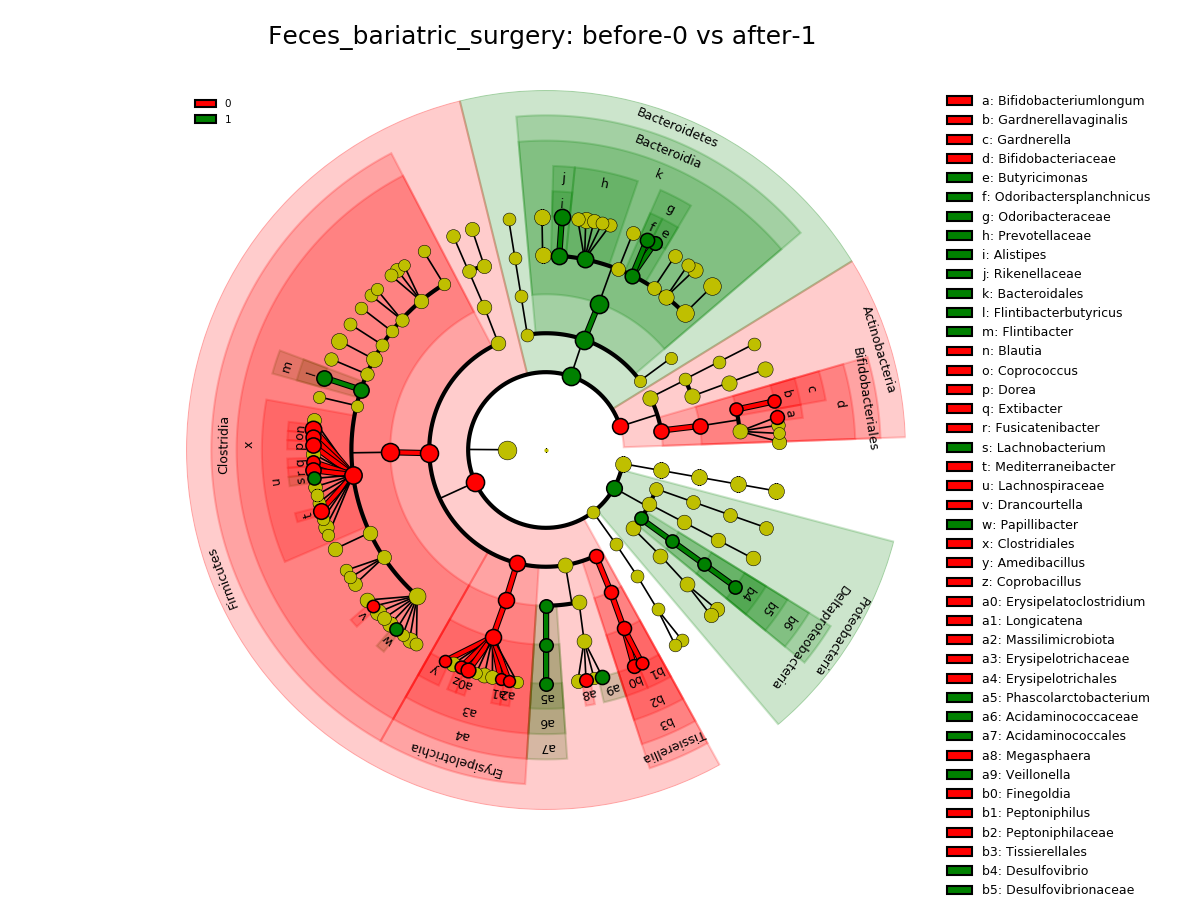


Appendix E. The composition of oral microbiota in successful and unsuccessful group before and after the surgery.


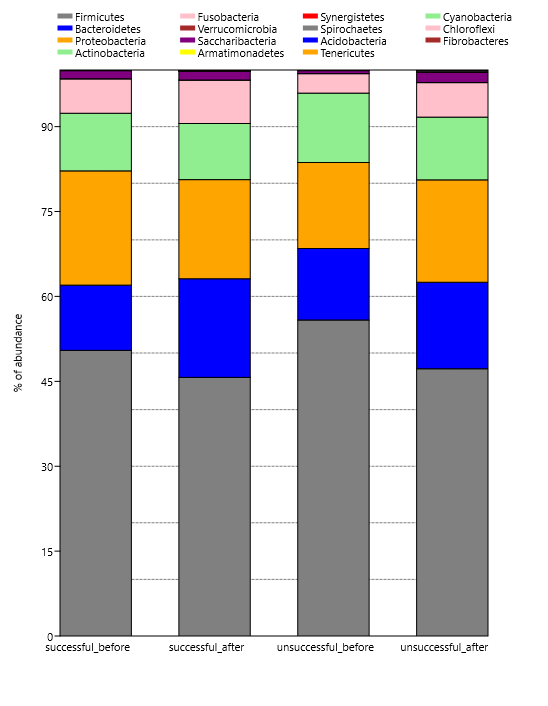


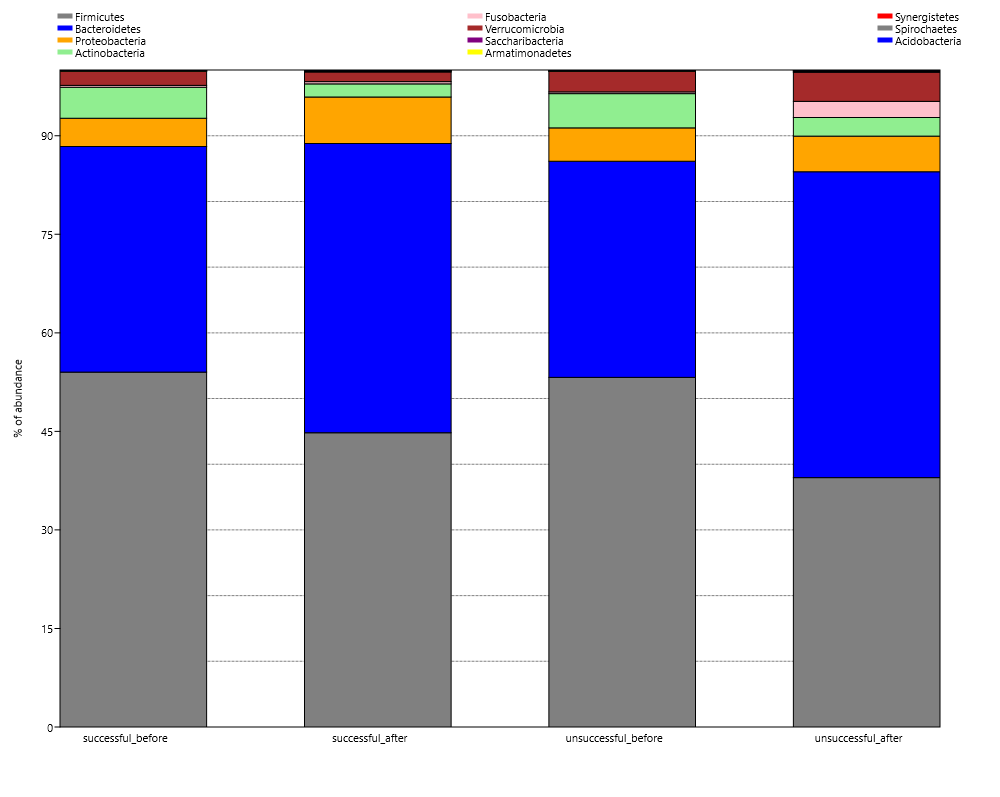
Appendix F. The composition of intestinal microbiota in successful and unsuccessful group before and after the surgery.

Appendix G. Differences in preoperative and postoperative oral microbiota among patients undergoing SG (0 – preoperative microbiota; 1 – postoperative microbiota).


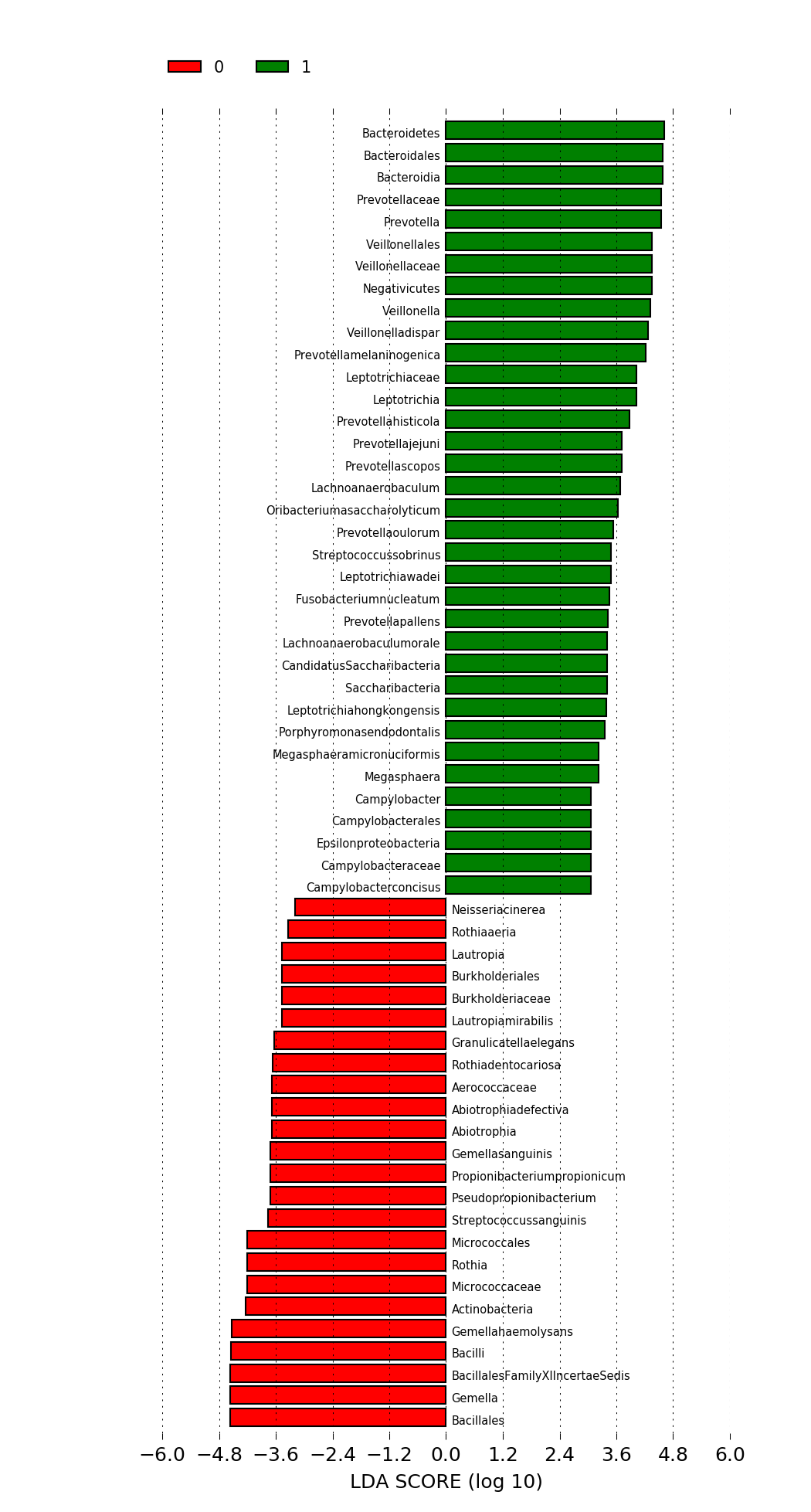


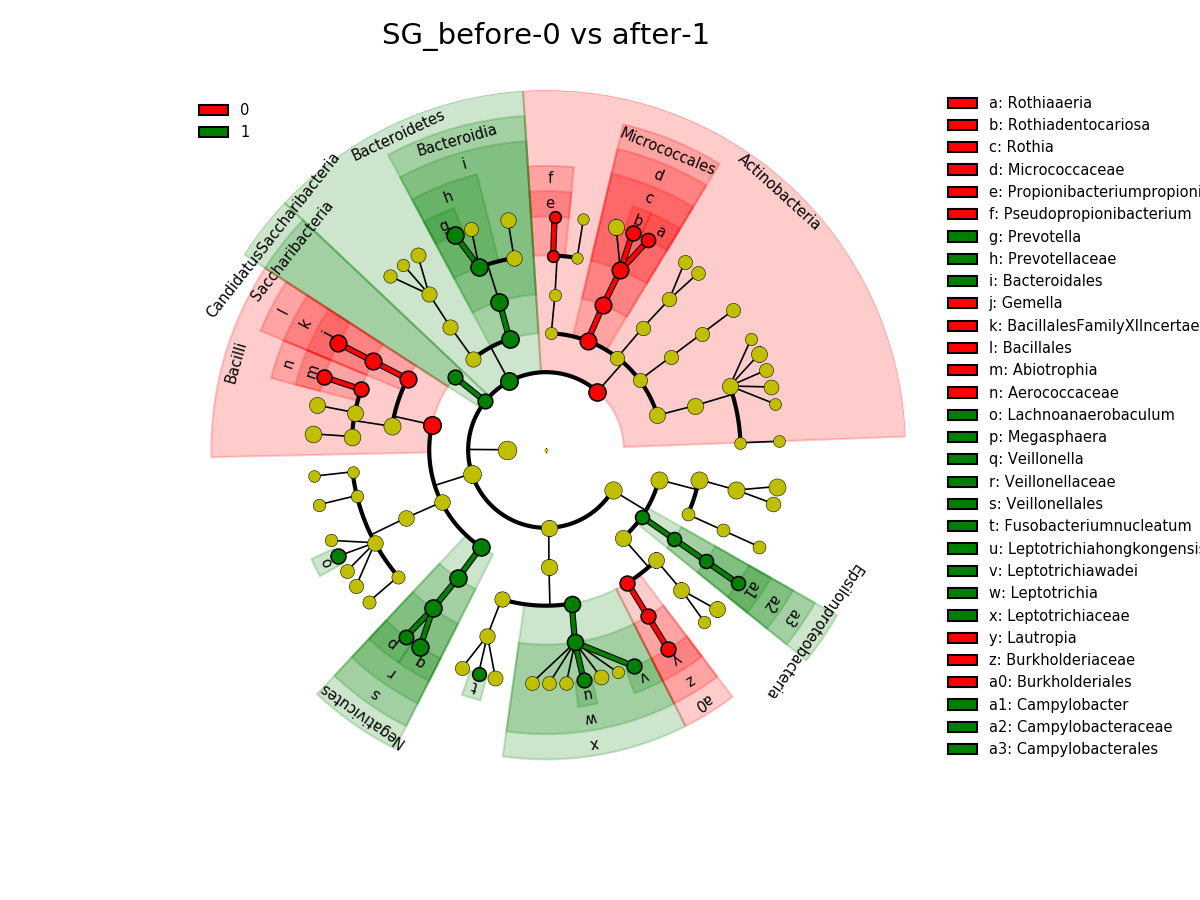


Appendix H. Differences in preoperative and postoperative intestinal microbiota among patients undergoing SG (0 – preoperative microbiota; 1 – postoperative microbiota).

**
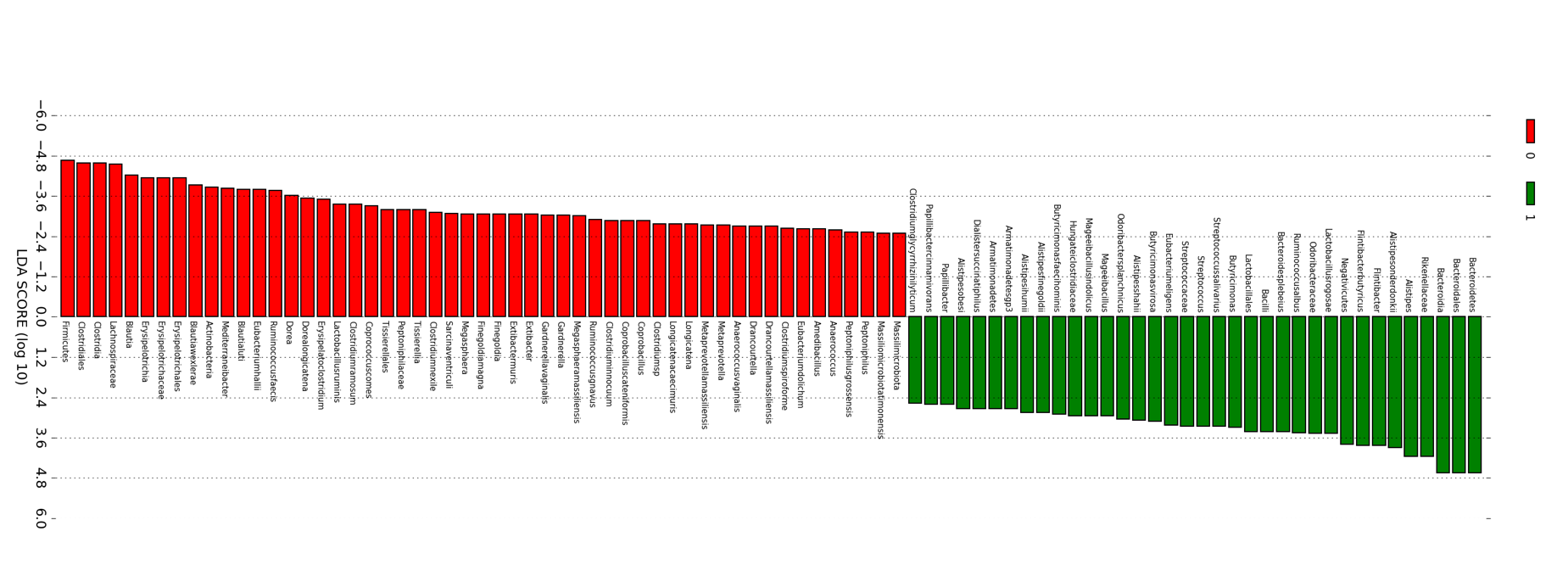
**


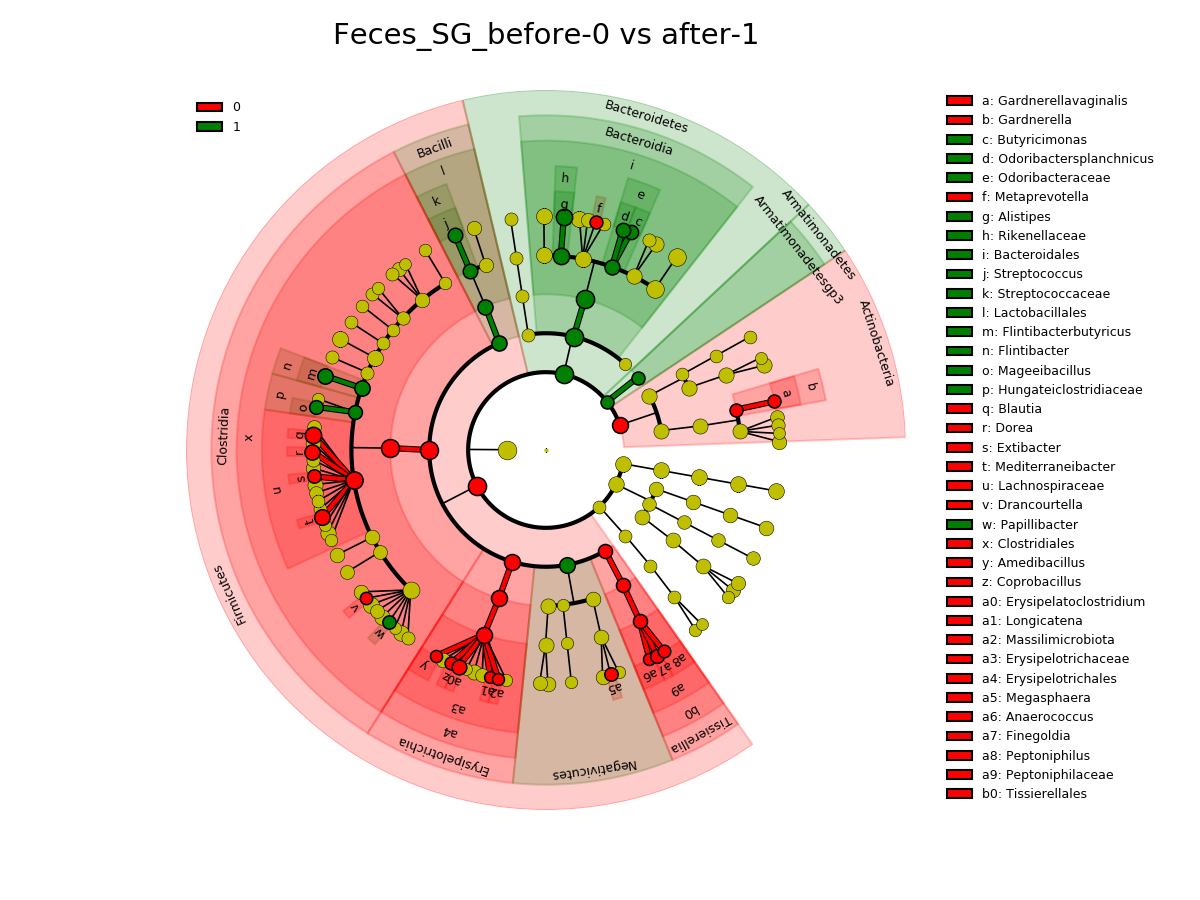


Appendix I. Differences in preoperative and postoperative oral microbiota among patients undergoing RYGB (0 – preoperative microbiota; 1 – postoperative microbiota).


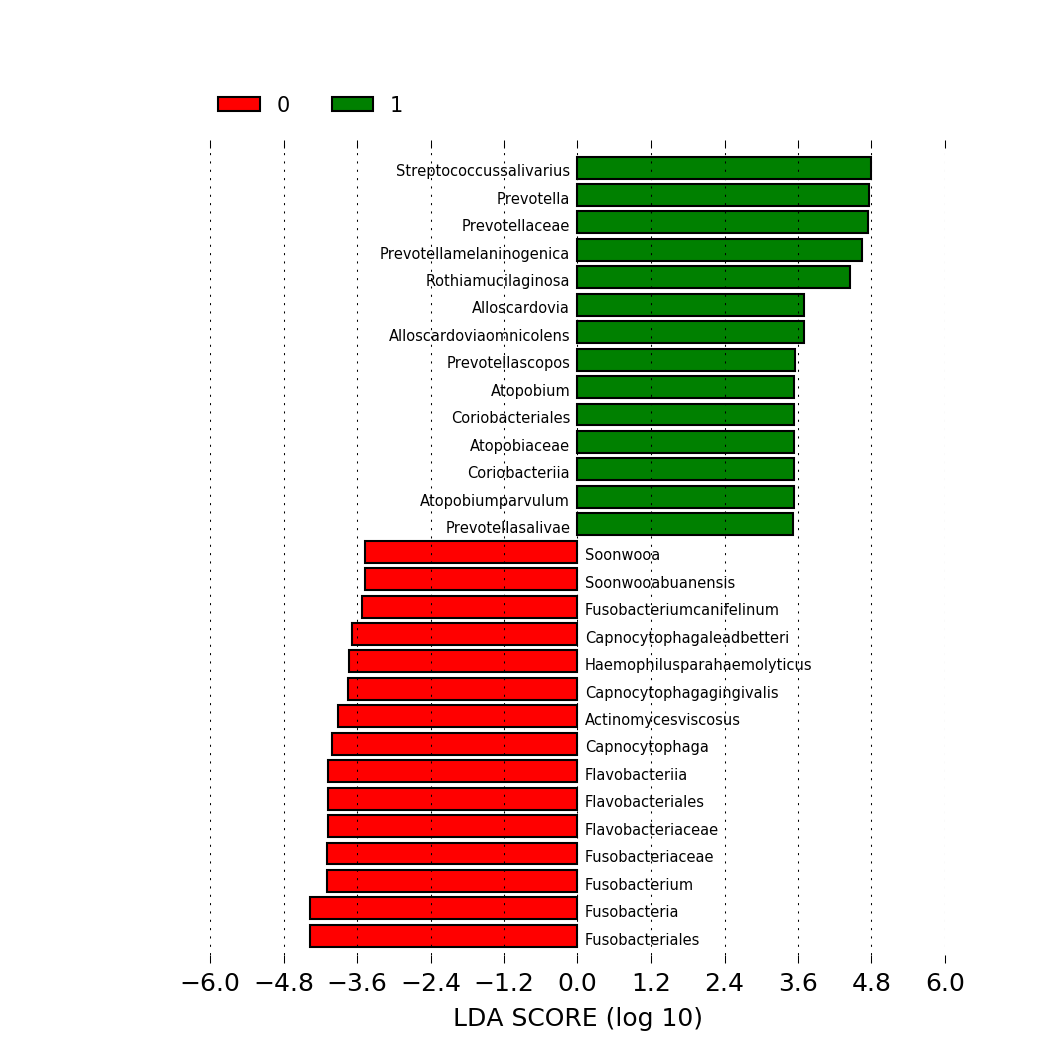

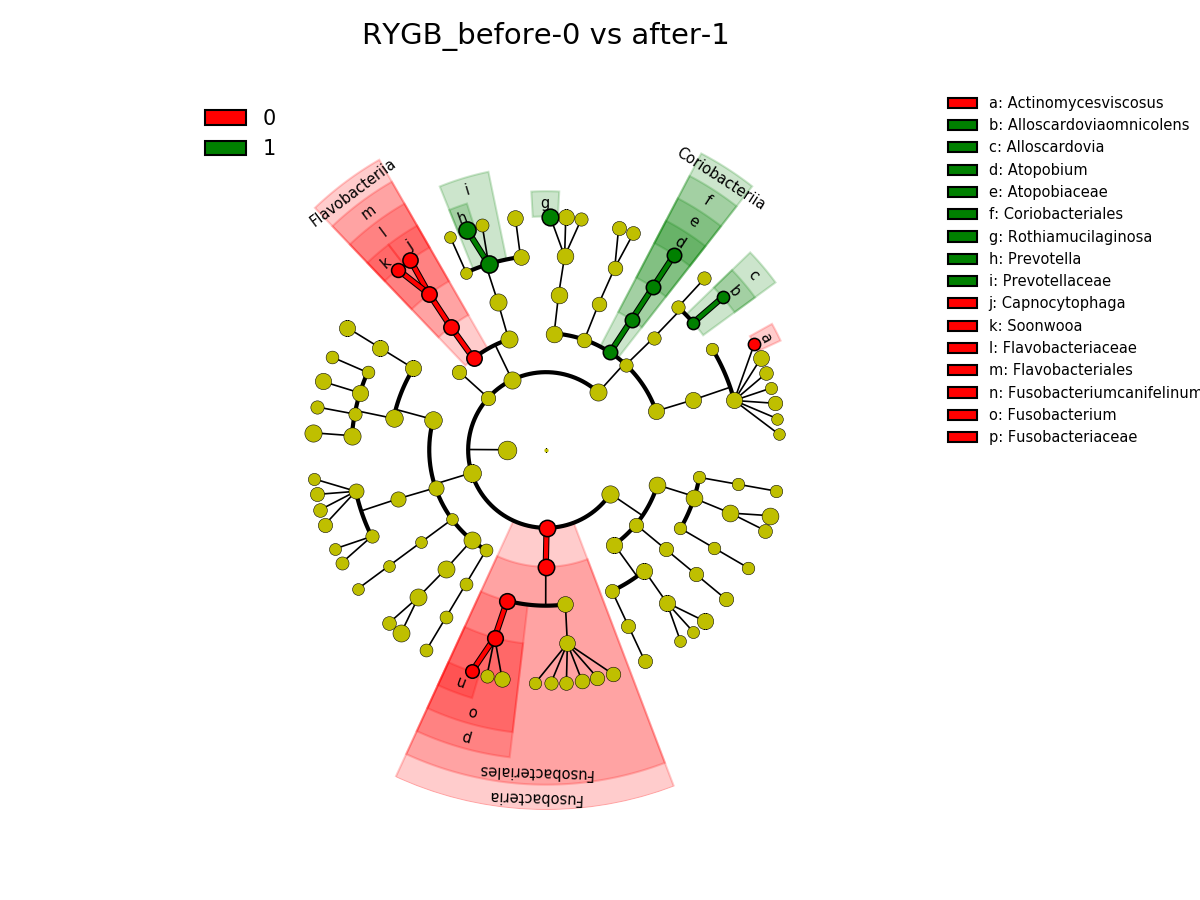


Appendix J. Differences in preoperative and postoperative intestinal microbiota among patients undergoing RYGB (0 – preoperative microbiota; 1 – postoperative microbiota).


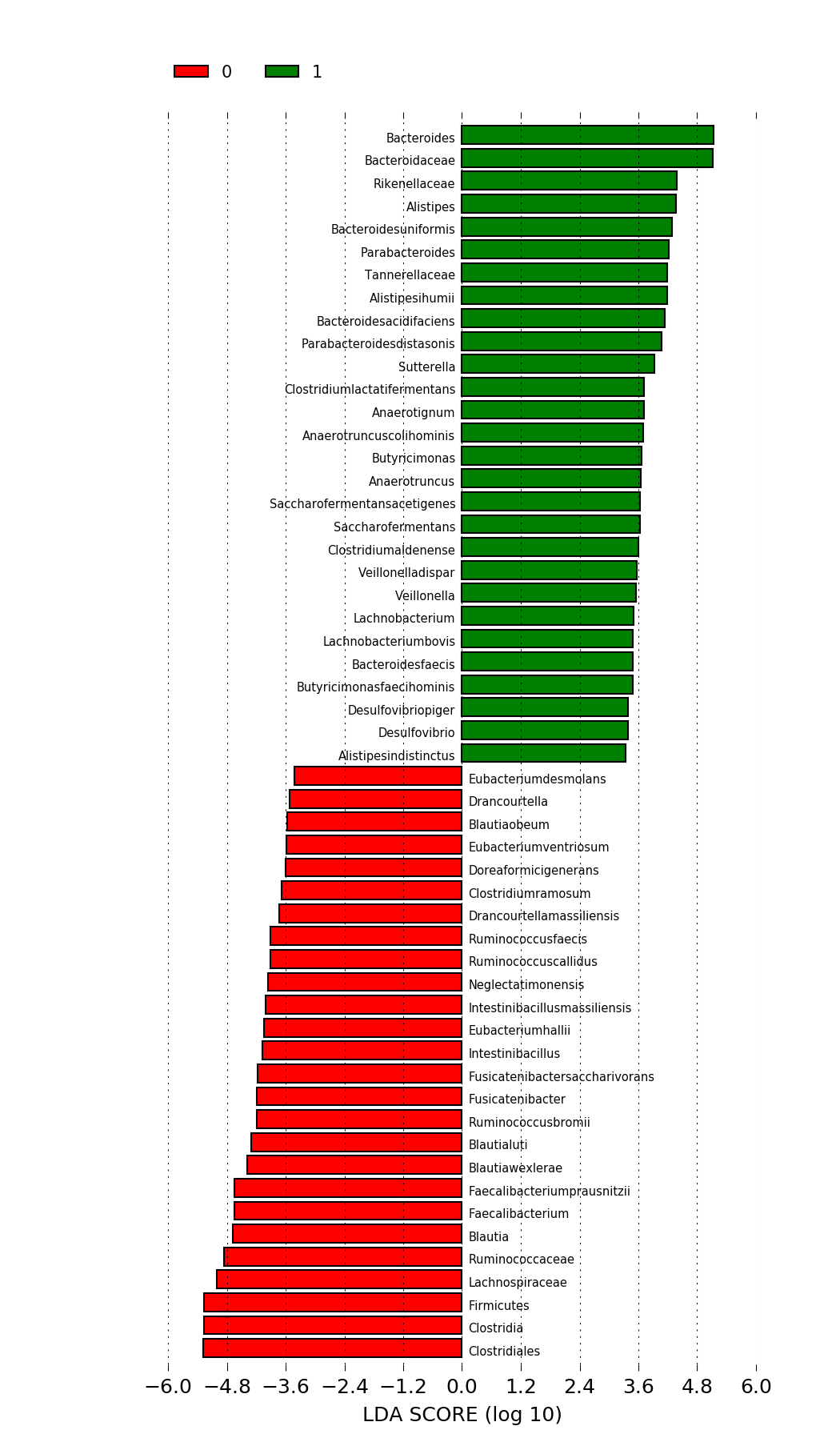

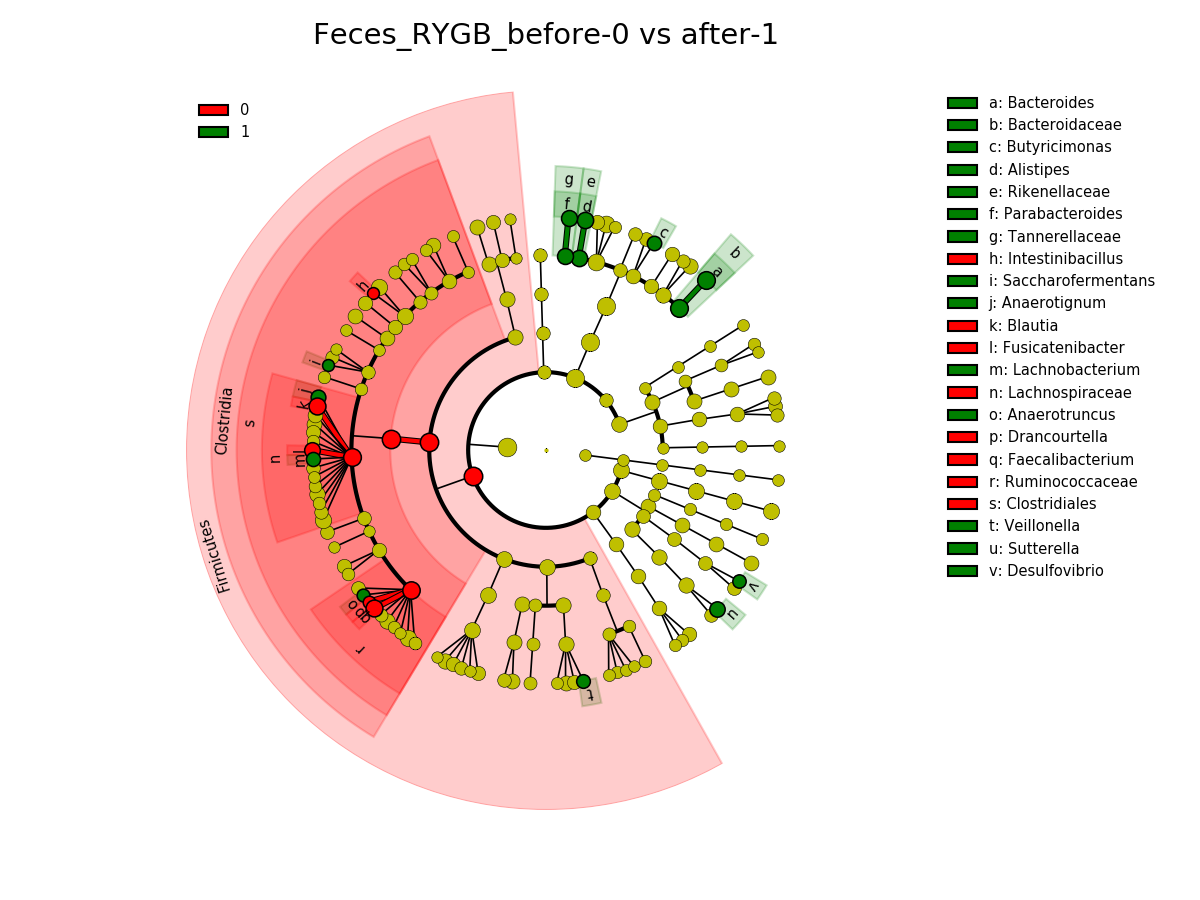


Appendix K. Differences in preoperative and postoperative oral microbiota among patients achieving successful outcomes after bariatric surgery (0 – preoperative microbiota; 1 – postoperative microbiota).


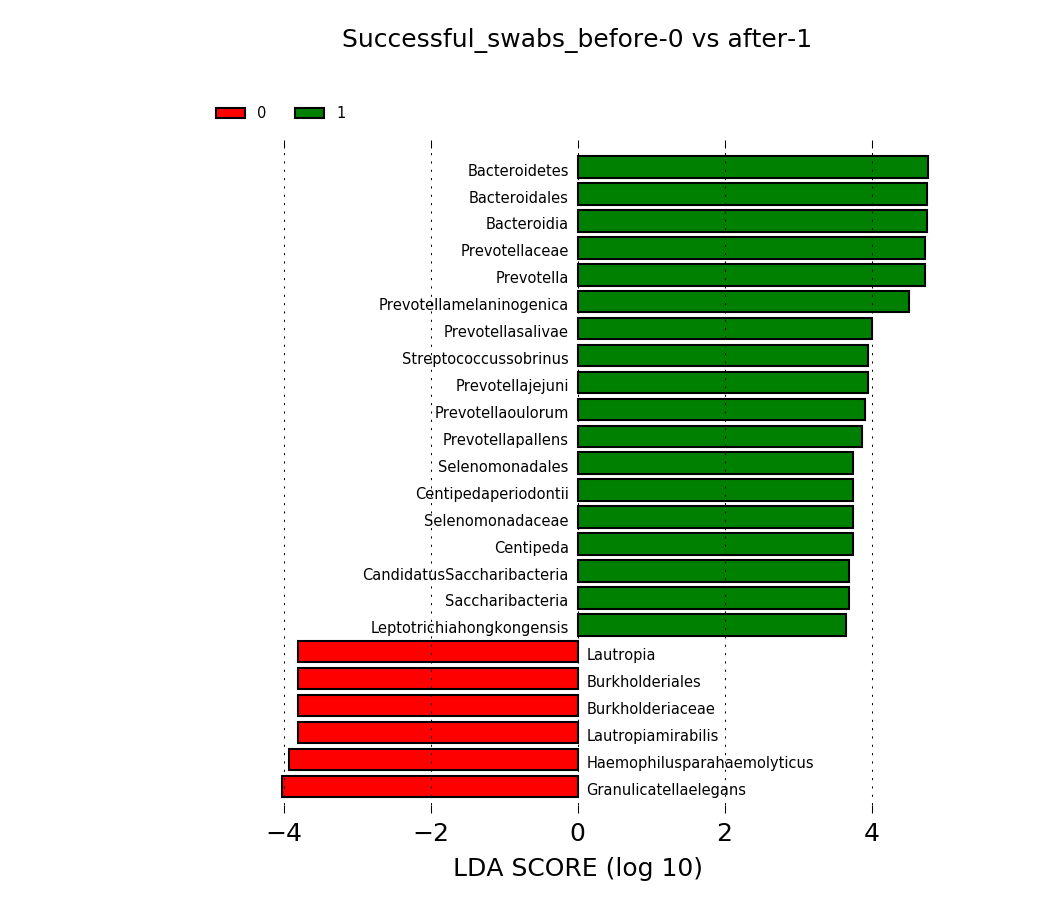

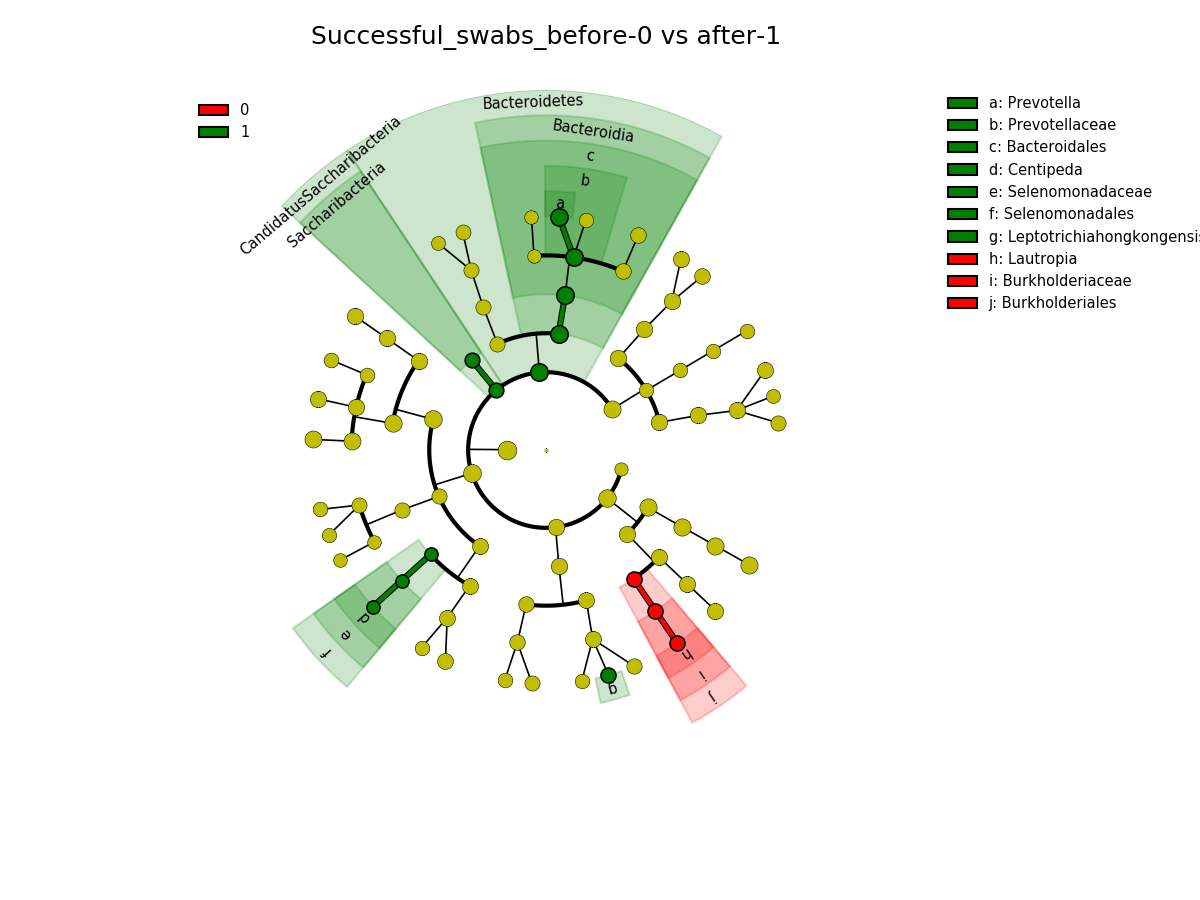


Appendix L. Differences in preoperative and postoperative intestinal microbiota among patients achieving successful outcomes after bariatric surgery (0 – preoperative microbiota; 1 – postoperative microbiota).
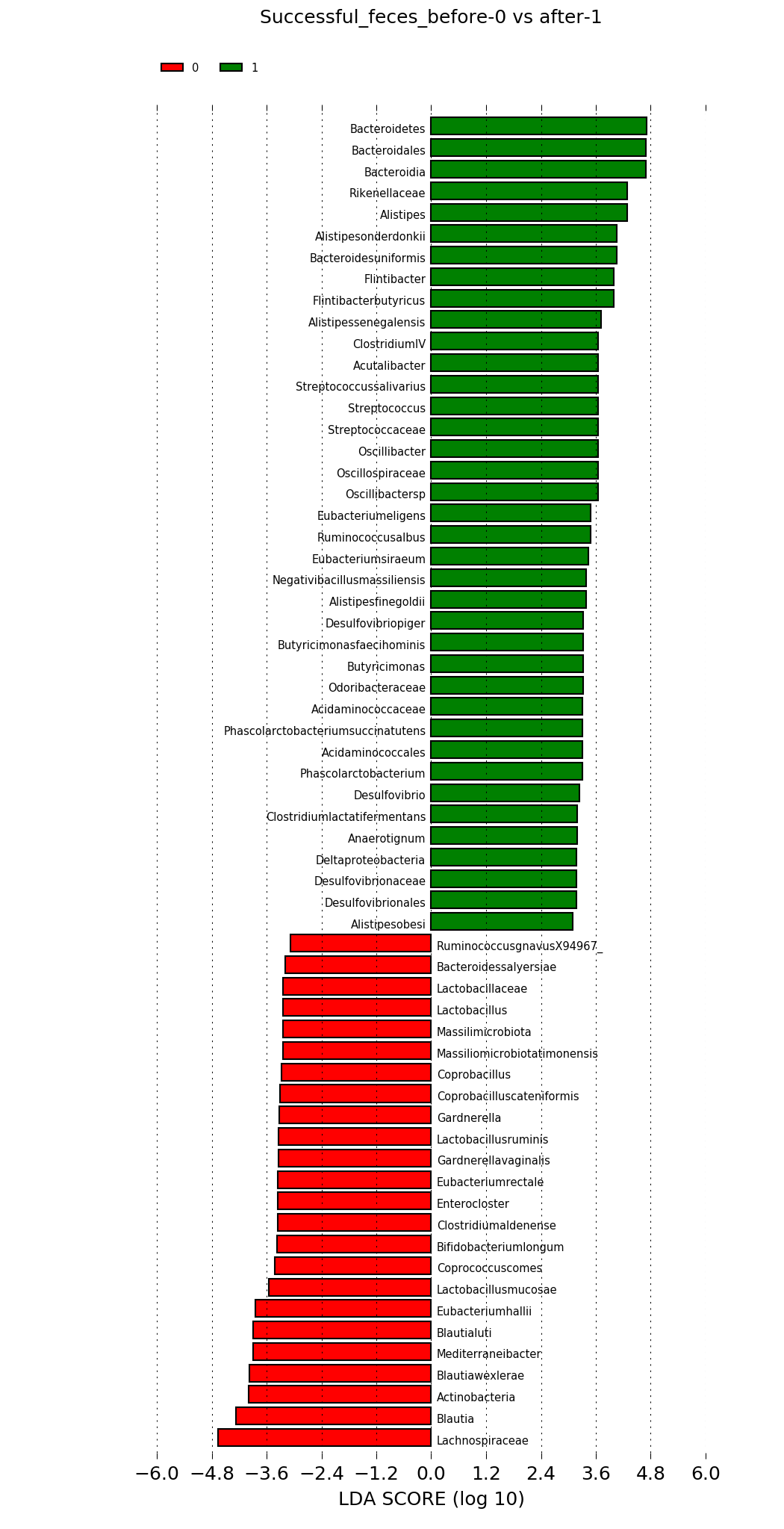


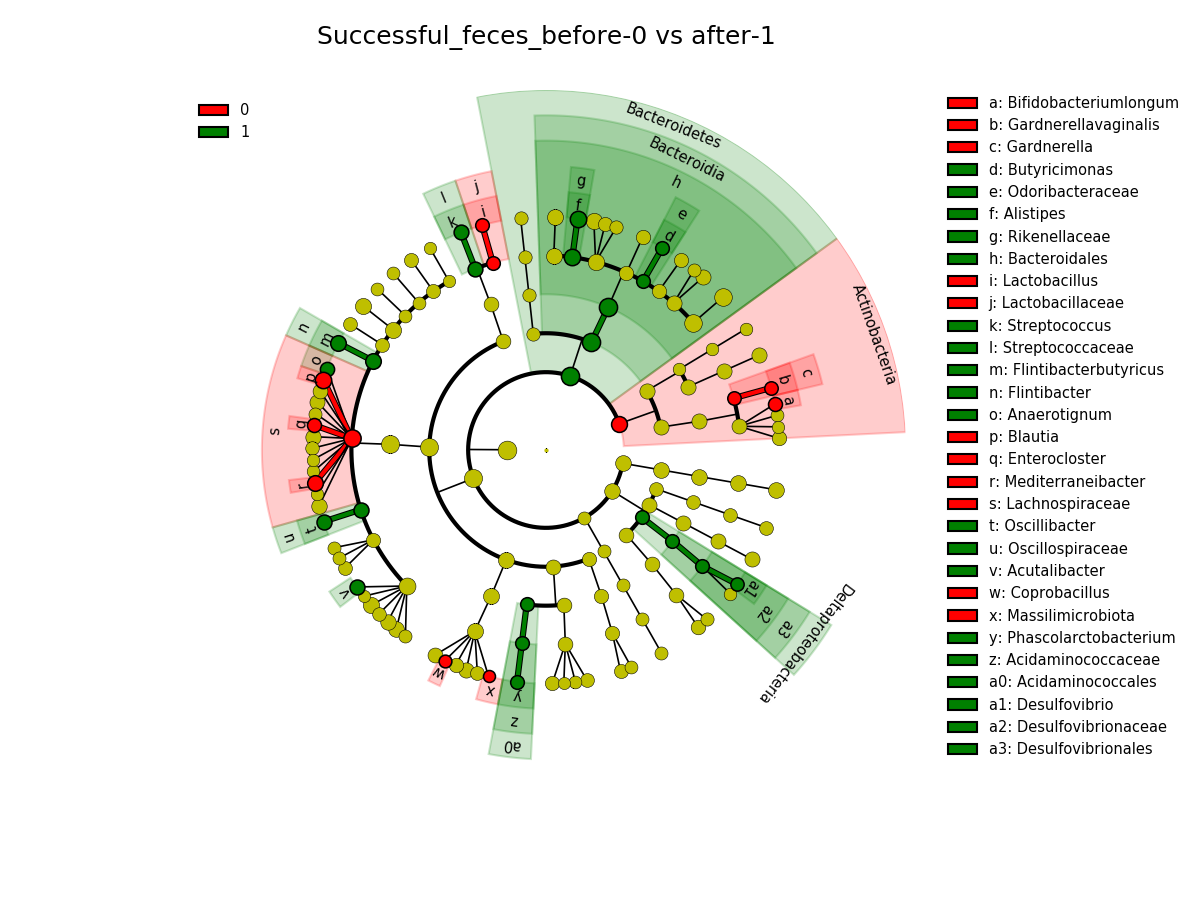


Appendix M. Differences in preoperative and postoperative oral microbiota among patients achieving unsuccessful outcomes after bariatric surgery (0 – preoperative microbiota; 1 – postoperative microbiota).


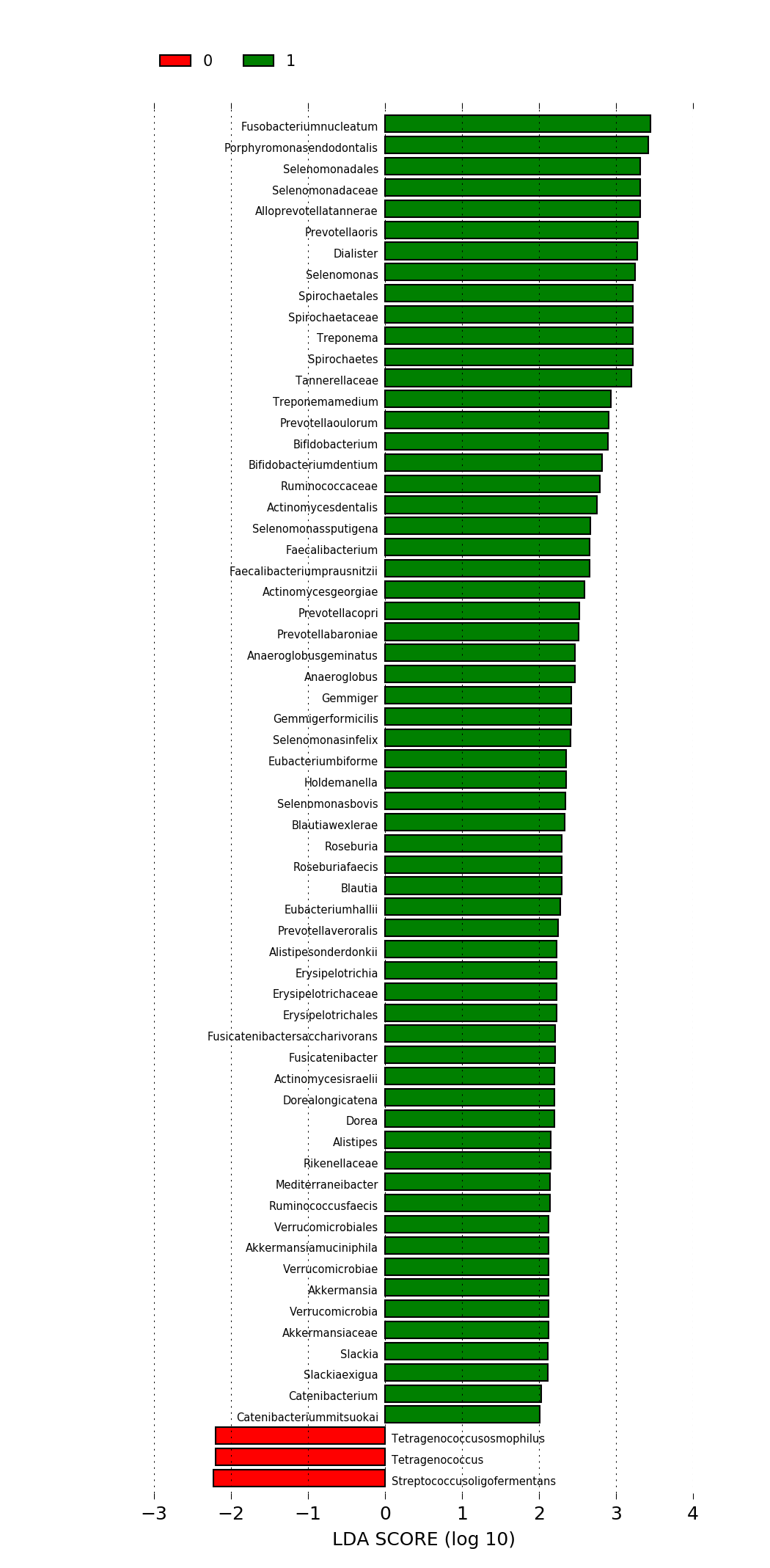


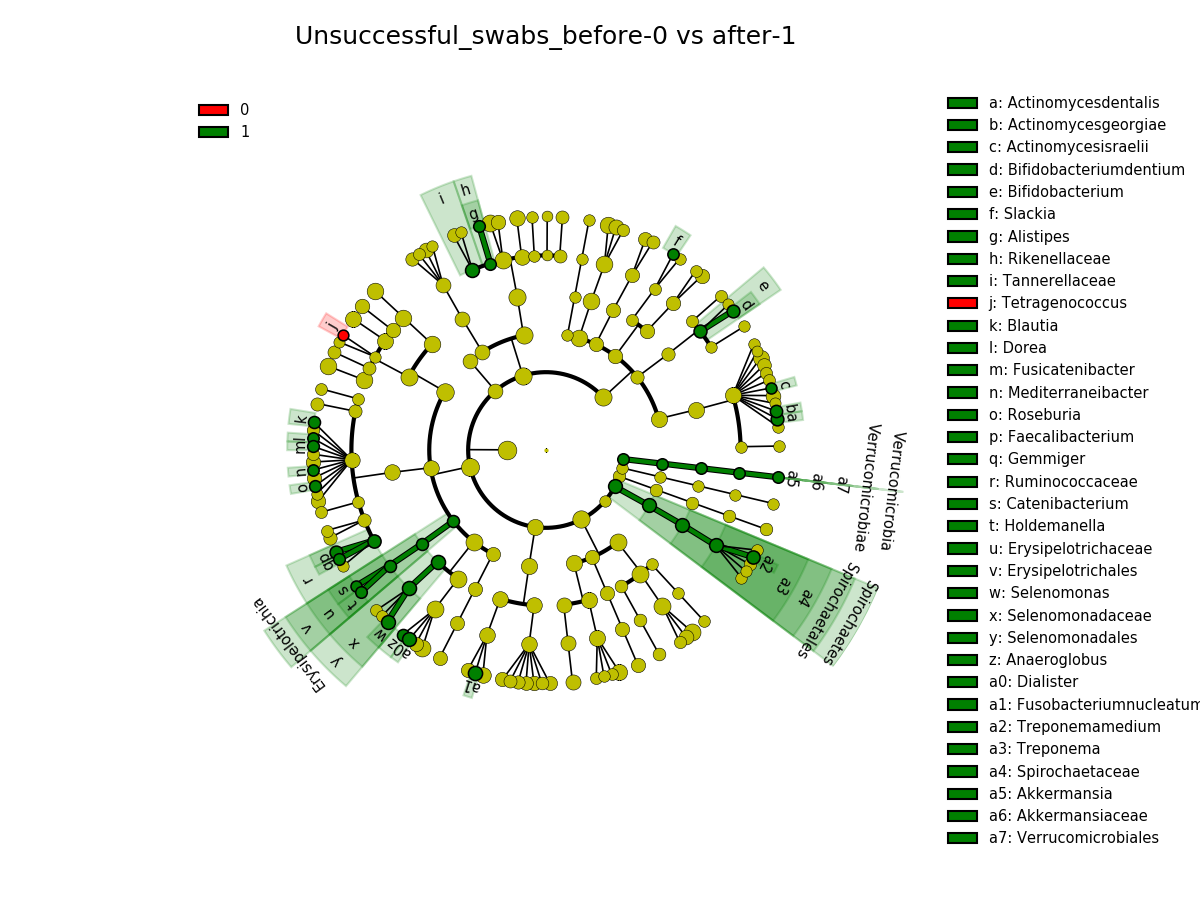


Appendix N. Differences in preoperative and postoperative intestinal microbiota among patients achieving unsuccessful outcomes after bariatric surgery (0 – preoperative microbiota; 1 – postoperative microbiota).


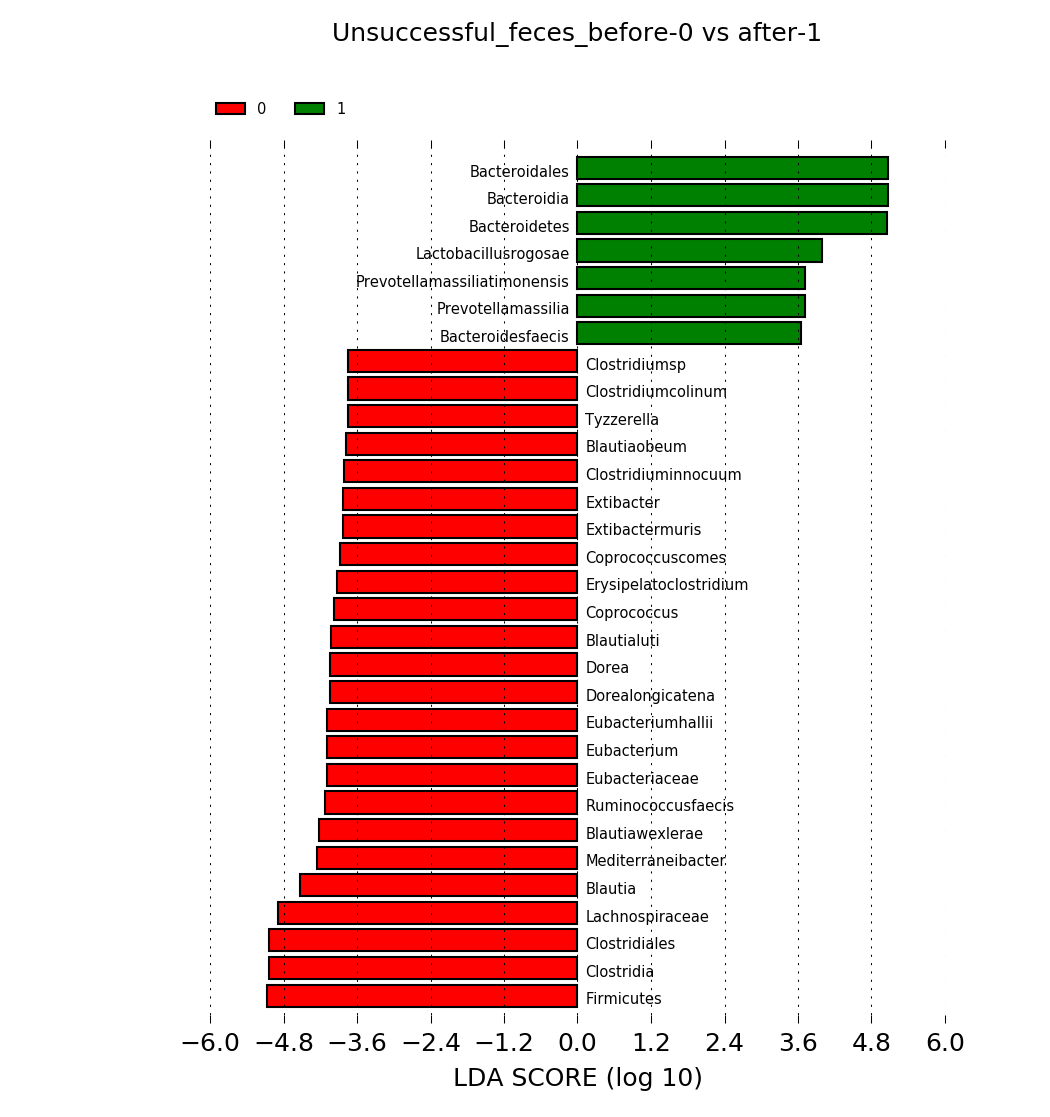

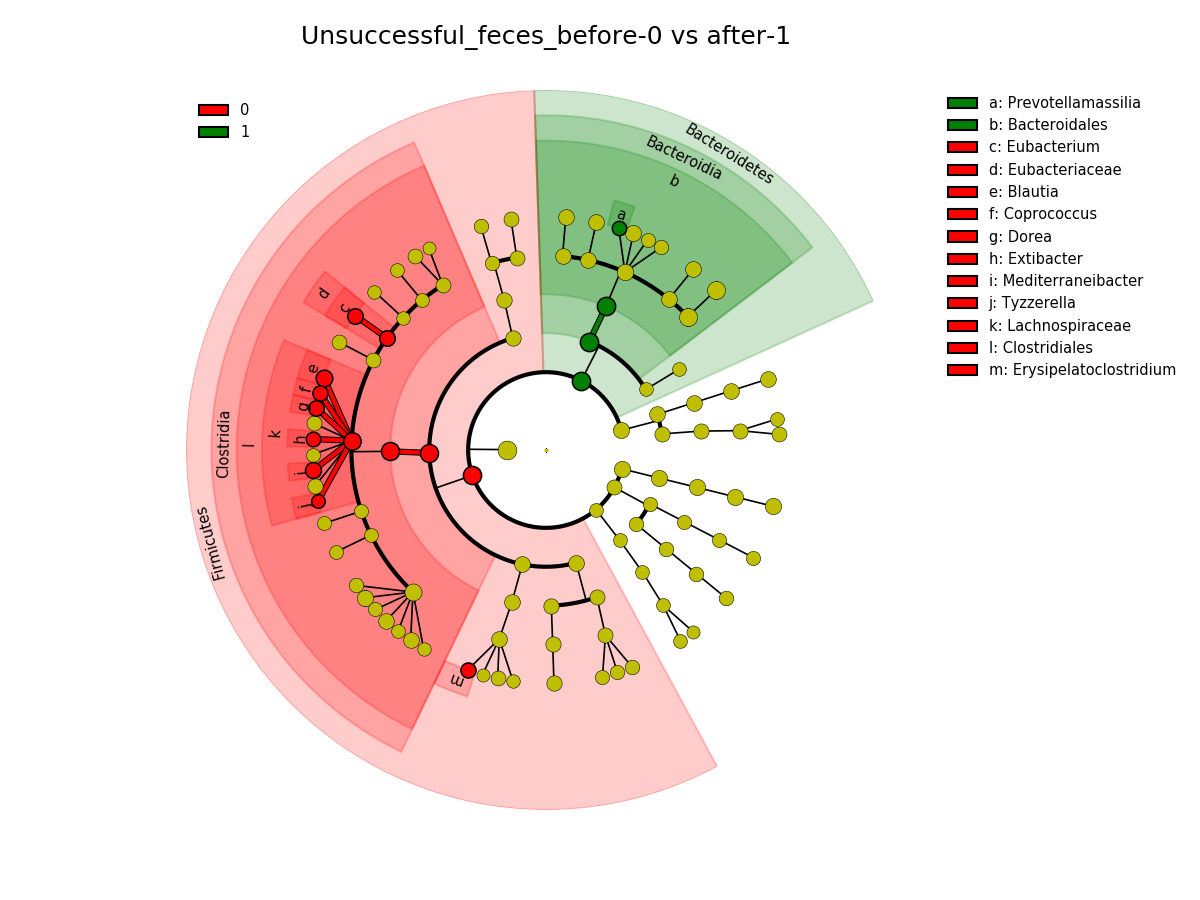

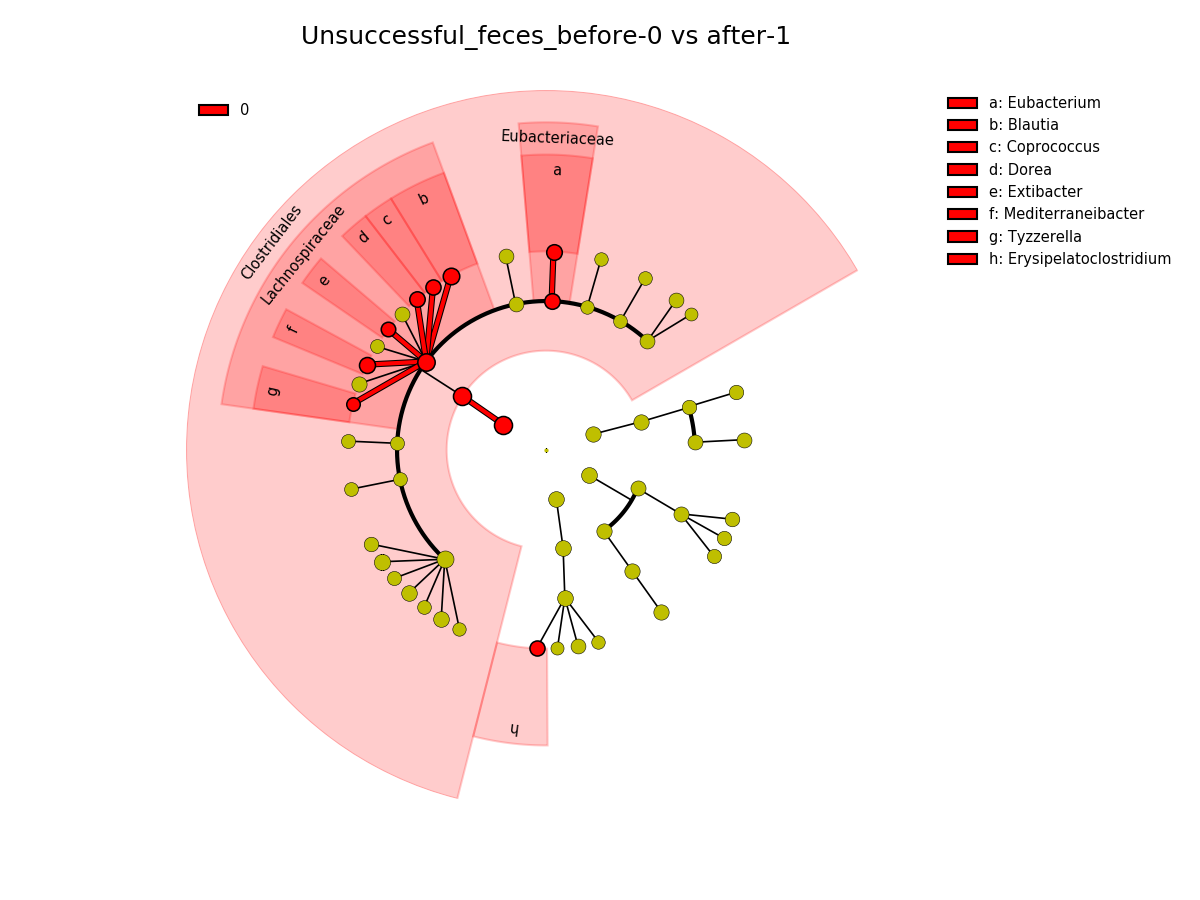

Supplement: Supplementary file 1 — Supplementary file1 (DOCX 4363 KB) [file 11695_2022_5954_MOESM1_ESM.docx]
